# Construction of a Prognostic Risk Model for *Helicobacter pylori* Infection in Gastric Cancer Patients and Immunological Analysis

**DOI:** 10.1002/cnr2.70511

**Published:** 2026-04-02

**Authors:** Zhiying Tian, Miao Su, Bin Yang, Zhaoyun Zhang, Li Zhang

**Affiliations:** ^1^ Department of Gastroenterology People's Hospital of Hengshui Hengshui Hebei China; ^2^ Department of Cardiovascular Medicine People's Hospital of Hengshui Hengshui Hebei China

**Keywords:** gastric cancer, *Helicobacter pylori*, immune infiltration, prognostic model, TCGA‐STAD

## Abstract

**Background:**

Gastric carcinoma poses a significant global health challenge, often diagnosed late due to its similarity to chronic gastric conditions. *Helicobacter pylori* (Hp) infection plays a crucial role in gastric carcinogenesis through inflammation and the release of virulent products.

**Aim:**

This study aimed to identify Hp infection‐related genes associated with gastric cancer prognosis and to develop a prognostic risk model that can predict patient outcomes, characterize the tumor immune microenvironment, and evaluate potential responses to immunotherapy.

**Methods and Results:**

In this study, multiple GEO datasets with Hp infection were analyzed to identify genes associated with gastric cancer prognosis. A predictive risk scoring model comprising nine genes related to Hp infection and gastric cancer prognosis was constructed and validated. The prognostic model demonstrated its efficacy in predicting prognosis, correlating with clinical characteristics, functional enrichment, immune cell infiltration, genomic mutations, and immune regulator expression. Additionally, analysis of immune therapy response suggested the potential prognostic effect of markers in gastric cancer immune response.

**Conclusions:**

These findings offer valuable insights into gastric cancer diagnosis and targeted therapy, paving the way for improved patient outcomes.

**Abbreviations**
AUC: area under curve
CAF: cancer‐associated fibroblast
CAG: chronic atrophic gastritis
DGE: differentially expressed gene
EGC: early gastric cancer
FC: fold change
FDR: false discovery rate
GEO: Gene Expression Omnibus
GO: Gene Ontology
GSEA: Gene set enrichment analysis
Hp:
*Helicobacter pylori*

HR: hazard ratio
IM: intestinal metaplasia
IPS: immunophenoscore
KEGG: Kyoto Encyclopedia of Genes and Genomes
KM: Kaplan–Meier
MDSC: myeloid‐derived suppressor cell
NAG: non‐atrophic gastritis
NES: normalized enrichment score
NK cells: natural killer cells
PCA: principal component analysis
ROC: receiver operating characteristic
TCGA‐STAD: The Cancer Genome Atlas Stomach Adenocarcinoma
TIDE: Tumor Immune Dysfunction and Exclusion
TMB: Tumor Mutation Burden

## 1 Introduction

Gastric carcinoma, a malignant tumor originating from the epithelium of the gastric mucosa, ranks fifth most common cancer globally and stands as the third leading cause of cancer‐related mortality [1, 2]. Its manifestations can mimic those of chronic gastric ailments like gastritis and gastric ulcers, potentially leading to delayed diagnosis. Consequently, early detection rates for gastric carcinoma remain low, resulting in diagnoses at advanced stages and missing the optimal window for surgical intervention.

*Helicobacter pylori* (Hp) infection, known for converting nitrate to nitrite and nitrosamine, is closely linked to the majority of cancer cases. Chronic Hp infection triggers gastric mucosal inflammation, accelerating epithelial cell proliferation and ultimately leading to gastric carcinoma [3]. Virulent Hp products, particularly *CagA* and *VacA*, may possess carcinogenic properties, evident from a higher prevalence of anti‐*CagA* antibodies in gastric carcinoma patients. Hp infection activates pro‐inflammatory signaling pathways, fostering gastric inflammation and carcinogenesis, making it a primary risk factor for gastric carcinoma [4].

In this study, we analyzed multiple public datasets with Hp infection, identifying differentially expressed genes (DGEs) between infected and uninfected gastric tissue (Figure S1). Through single‐factor Cox analysis on shared DGEs, we pinpointed biomarkers significantly associated with gastric cancer prognosis in The Cancer Genome Atlas Stomach Adenocarcinoma (TCGA‐STAD) cohort. Subsequently, we constructed and validated a predictive risk scoring model comprising nine genes related to gastric cancer prognosis due to Hp infection across TCGA and the Gene Expression Omnibus (GEO) validation sets. Our comprehensive evaluation confirmed the risk score as an independent prognostic factor, significantly correlated with clinical characteristics, HALLMARK functional enrichment, immune cell infiltration, genomic mutations, and immune regulator expression in gastric cancer. Analysis of immune therapy response indicated the potential prognostic effect of markers in gastric cancer immune response. Finally, we explored the expression levels of prognostic genes across immune cell types in single‐cell data. Our proposed prognostic model holds promise for diagnosing gastric cancer and guiding targeted therapy efforts.

## 2 Materials and Methods

### 2.1 Datasets Download and Preprocessing

A comprehensive workflow of this study is illustrated in Figure S1. Briefly, the analysis commenced with the acquisition and preprocessing of transcriptomic datasets from both microarray and RNA sequencing platforms.

Microarray gene expression datasets from Hp‐infected gastric cancer patients and uninfected controls were sourced from GEO (GSE60662, GSE27411, and GSE60427) [3, 5, 6]. Raw gene chip datasets were downloaded, with the highest value probe representing gene expression for genes with multiple probes. Background correction and normalization were applied, and probe names were matched to their corresponding gene symbols.

RNA expression data of 388 TCGA‐STAD samples were obtained from the Genomic Data Commons (GDC) portal via the R package TCGAbiolinks [7]. CNV profiles and mutation profiles for TCGA‐STAD patients were also extracted. Survival data were sourced from the TCGA Pan‐Cancer Clinical Data Resource [8]. For external validation of the prognostic model, three additional GEO datasets for gastric cancer samples were used: GSE26901 (*n* = 76), GSE84426 (*n* = 109), and GSE13861 (*n* = 62) [9, 10, 11]. A dataset of metastatic urothelial cancer with PD‐L1 immune therapy outcomes was also obtained. A single‐cell dataset of gastric cancer (GSE134520) was used to evaluate cell type‐specific gene expression of prognostic signatures [12].

### 2.2 Differential Gene Expression Analysis

Differential gene expression analysis between Hp‐infected individuals and controls was performed using the R package Limma on datasets GSE60662, GSE27411, and GSE60427 [13]. DEGs were defined as those with a *p* < 0.05 and an absolute log2(Fold Change) > 0.585. Common DEGs from the three datasets were identified by overlapping the results.

### 2.3 Construction and Validation of Prognostic Model

Univariate Cox analysis calculated the hazard ratio (HR) for each gene based on survival data. HRs with *p* < 0.05 were considered significant for gastric cancer prognosis. Significant HR genes were further filtered using the Lasso model with STAD data, divided into 10 subsamples, with 7 subsamples randomly selected as the training set. The optimal lambda (lambda = 9) was chosen using the lowest partial likelihood deviance with a random seed of 1210, resulting in 9 genes. These genes' survival analysis was performed on STAD patients from the TCGA cohort, with samples divided into high and low expression groups based on the median expression value. Kaplan–Meier (KM) survival curves and log‐rank tests assessed survival differences, with *p* < 0.05 considered significant. The associated genes were selected as prognostic signatures. These genes were used to construct the risk model for gastric cancer prediction based on their coefficient from lasso regression model. The risk model was present as

$$\text{Riskscore}=\sum_{i=0}^{n}\mathrm{\beta i}\times \mathrm{\chi i},$$

where βi represents the loading and χi represents the expression of gene *i*.

The risk model was validated using the training sets, the entire STAD set and three external GEO datasets (GSE26901, GSE84426, GSE13861). Receiver operating characteristic (ROC) curve analysis evaluated the model using the R package timeROC [14].

### 2.4 Gene Set Enrichment Analysis (GSEA)

Single‐gene GSEA was performed using STAD expression profiles for prognostic signatures based on gene list ranking by Pearson correlation coefficients between each gene and genes associated with specific functional gene sets including HALLMARK sets from MsigDB database, curated immune functional gene sets and tumor and immune system interaction gene sets from TISIDB database [15, 16, 17, 18].

The GSEA analysis for DGEs was carried out with Gene Ontology (GO) and Kyoto Encyclopedia of Genes and Genomes (KEGG) databases. Significantly enriched terms were filtered with a *p* < 0.05, and the top 10 enriched pathways ranked by normalized enrichment scores (NESs) were presented using the R package clusterProfiler [19].

### 2.5 Immune Infiltration Analysis

Four methods (CIBERSORT, ESTIMATE, TIMER, and ssGSEA) from the IOBR package were used to estimate immune cell profiling and calculate infiltration scores for 22 types of immune cells [20]. The correlation between prognostic signature genes and immune cells was determined using Spearman's correlation analysis.

CIBERSORT algorithm is a method to characterize cell composition based on the gene expression profiles of complex tissues. It utilizes the leukocyte signature matrix LM22, composed of 547 genes, to distinguish 22 types of immune cells, including myeloid subpopulations, natural killer (NK) cells, plasma cells, naïve and memory B cells, and 7 types of T cells. Using CIBERSORT combined with the LM22 feature matrix, it estimates the proportions of the 22 cell phenotypes in a sample, with the sum of all immune cell type proportions in each sample equaling 1. ESTIMATE algorithm calculates immune scores, tumor purity, stromal scores, and ESTIMATE scores. TIMER estimates the abundance of 6 immune cell types (B cells, CD4+ T cells, CD8+ T cells, neutrophils, macrophages, and dendritic cells) in 32 types of cancers using a deconvolution method. Based on a set of marker genes for 28 immune cells, the enrichment score of each sample's 28 immune cell gene set is evaluated using the R package GSVA's ssGSEA, indicating the infiltration level of the 28 immune cell types. The correlation between the prognostic signature genes and immune cells was determined using Spearman's correlation analysis.

### 2.6 Genome Single Nuclei Variation (SNV) Analysis

Based on the somatic mutation profiles from TCGA‐STAD cohort, SNV mutation differences between high and low‐risk groups defined by the median risk score were presented using oncoplots generated from the R package maftools [21]. The concurrence degree of top 10 mutated genes in both groups was calculated with the function somaticInteractions(), and tumor mutation burden was compared between high and low‐risk groups using the function mafCompare().

### 2.7 Immunotherapy Response Prediction

Immunophenoscore (IPS) scores for gastric cancer were downloaded from the TCIA database to analyze differences in immunogenicity between high and low‐risk groups and predict the response of various tumor types to immunotherapy. Tumor immune dysfunction and exclusion scores were calculated using the TIDE algorithm (TIDE: http://tide.dfci.harvard.edu) [22] to infer patients' response to immune checkpoint blockade therapy. This algorithm is based on pre‐treatment expression profiles of tumors, scoring multiple published transcriptomic biomarkers to predict patients' response to immunotherapy. The TIDE score integrates T cell dysfunction and exclusion features, simulating tumor immune escape scenarios with varying levels of tumor‐infiltrating cytotoxic T cells, which proves significantly advantageous compared to other biomarkers.

### 2.8 Single Cell Transcriptome Analysis

The scRNA‐seq data downloaded from GSE134520 [12] were processed with R package Seurat (v5) for quality control and integration [23]. The dataset was originally generated from 13 gastric antral mucosa biopsies from nine patients with Non‐atrophic gastritis (NAG), Chronic atrophic gastritis (CAG), Intestinal metaplasia (IM), or early gastric cancer (EGC). Specifically, for each sample, the low‐quality cells were pre‐filtered by only keeping those with at least detected 200 genes. After merging of all samples, low quality cells and possible doublets were further filtered by keeping cells with detected genes between 300 and 600, UMIs between 300 and 40 000, and the mitochondrial gene ratio lower than 20% and read ratio of red cells lower than 20%. The integrated data was normalized and scaled with top 2K highly variable genes. Principal Component Analysis (PCA) was performed for dimension reduction and the top 20 components were picked from the ElbowPlot function for running UMAP. To remove the batch effect, the datasets were integrated with the RunHarmony function with batch effect correction, followed by identifying cell groups using the FindClusters function with a resolution of 0.8.

The cell type annotation of clusters was carried out with the SingleR package using BlueprintEncodeData and HumanPrimaryCellAtlasData as reference databases [24]. The algorithm iteratively calculated the Spearman correlation coefficient of variable genes in single cells with each sample in the reference dataset. The 80th percentile of correlation coefficients of multiple reference samples under the same cell type was used as the score for annotating a single cell to this cell type. Reference cell types with a maximum score difference within 0.05 were retained until only two cell types remained, where the known cell type with the highest correlation score was retained for annotating the cells. After the initial annotation of cell types with reference databases, the markers of each cell type were identified with the FindAllMarkers function, setting min.pct at 0.25 and logfc.threshold at 0.25. A threshold of avg_log2FC > 0.5 and p_val_adj < 0.05 was applied to filter significant markers. Finally, the annotation results from SingleR were further manually refined based on the marker genes from each annotated cluster with the marker reference from the TISCH database.

With the finalized cell types, the scores of prognostic signatures from each cell type were calculated with R package GSVA. Functions VlnPlot and DotPlot were employed to visualize the cell landscape and subpopulation gene expression distribution in different gastric cancer cell types.

### 2.9 Statistical Analysis

For statistical analysis, the Wilcoxon test was used to compare differences between two sample groups, while the Kruskal−Wallis test was used to compare differences between multiple sample groups. Specifically, “ns” indicated *p* > 0.05, “*” indicated *p* ≤ 0.05, “**” indicated *p* ≤ 0.01, “***” indicated *p* ≤ 0.001, and “****” indicated *p* ≤ 0.0001.

## 3 Results

### 3.1 Identification and Functional Annotation of Dysregulated Genes in Hp‐Infected Patients

**FIGURE 1:**
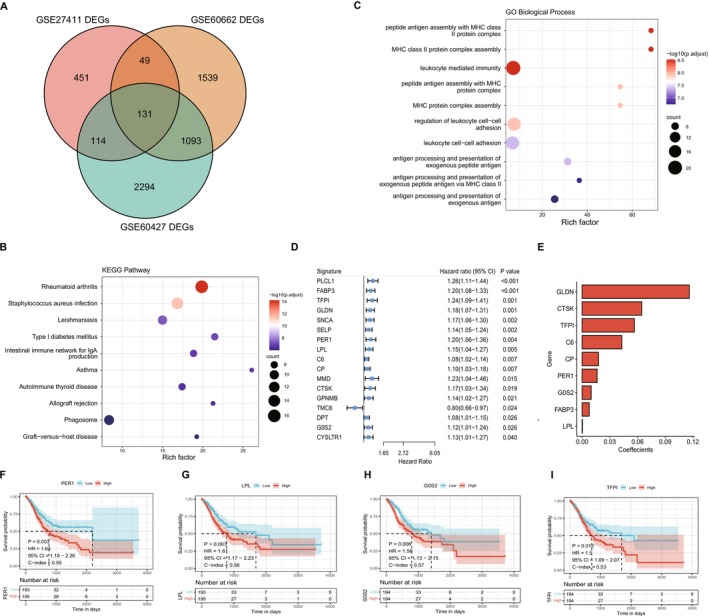
Identification and enrichment analysis of DEGs between Hp‐infected and uninfected tissues. (A) Venn diagram of DEGs from three datasets. (B, C) Enrichment analysis of genes associated with Hp infection for KEGG and GOBP (biological processes). The size of the dots represents the number of enriched DEGs, while the color represents the significance of the enrichment. (D) Forest plot showing risk estimation of each variable from single‐factor Cox analysis of risk model in TCGA‐STAD. (E) LASSO regression coefficients for the nine key prognostic factors. (F–I) Kaplan–Meier curves for model genes. Red represents high expression group and blue represents low expression group, and the *p* value indicates the significance of the survival curve difference.

To compare gene dysregulation in Hp‐infected gastric tissues versus uninfected controls, we curated three GEO datasets: GSE60662 (12 Hp‐infected vs. 4 controls), GSE27411 (12 Hp‐infected vs. 6 controls), and GSE60427 (24 Hp‐infected vs. 8 controls) (Table S1). Differential expression analysis was performed independently for each dataset. GSE60662 identified 2812 DEGs, with 1537 up‐regulated and 1275 down‐regulated in Hp‐infected samples (Figure S2A). GSE27411 detected 745 DEGs, including 440 up‐regulated and 305 down‐regulated (Figure S2B). GSE60427 observed 3632 DEGs, with 2085 up‐regulated and 1547 down‐regulated (Figure S2C). By intersecting the results from the three datasets, we identified 131 common DEGs for further analysis (Figure 1A).

To understand the functions and pathways of these dysregulated genes in gastric carcinoma, we performed pathway enrichment analyses on the 131 common DEGs (Table S2). The top 10 significant pathways (FDR < 0.05) for KEGG, GO: Biological Processes (BP), GO: Cellular Components (CC), and GO: Molecular Functions (MF) were presented (Figure 1B,C, Figure S2D,E). Results showed that DEGs were mainly involved in immune‐related pathways such as *Staphylococcus aureus* infection, type I diabetes, intestinal immune networks for *IgA* production, and transplant rejection. GO enrichment indicated that DEGs were associated with cellular immunity, assembly and processing of MHC class II‐related proteins, MHC class II receptor activity, antigen processing and presentation, immune receptor activity, chemotaxis factors, and other related processes. These findings suggest that Hp infection in gastric tissues primarily drives immune‐related alterations, particularly antigen presentation and T‐cell activation pathways. This is consistent with previous studies showing that Hp promotes chronic inflammation through MHC class II modulation and recruitment of immune cells, thereby facilitating gastric mucosal injury and carcinogenesis [3, 4]. The enrichment of chemotaxis and cytokine‐related processes further highlights the role of immune dysregulation as an early hallmark of Hp‐associated gastric cancer.

### 3.2 Identification of Prognostic Signature

The list of 131 commonly identified DEGs served as candidate genes for constructing a prognostic model in gastric cancer. To further assess their prognostic significance, single‐factor Cox analysis was conducted on each gene, resulting in 17 significant genes with *p* < 0.05 (Figure 1D). Among these, TMC6 exhibited a hazard ratio (HR) of 0.8 (95% CI: 0.66–0.97), indicating a lower risk in the Hp‐infected group compared to the control group; all other genes showed a higher risk with HR larger than 1 in gastric cancer.

The 17 genes underwent further screening for model construction using Lasso linear regression. Through random sampling, 7/10 of the overall TCGA‐STAD dataset (*n* = 388) were extracted as the training set (*n* = 271). Based on the training set, Lasso regression yielded an optimal lambda at 0.0372 via cross‐validation, resulting in a model of 9 prognostic signatures after shrinkage (Figure S2F,G): *GLDN*, *CTSK*, *TFP1*, *C6*, *CP*, *PER1*, *G0S2*, *FABP3* and *LPL* (Table S3). Their contributions to the prognostic model for gastric cancer were illustrated in Figure 1E. Furthermore, these 9 prognostic genes were evaluated through survival analysis individually. STAD patients were classified into high and low groups based on the median expression of each gene, revealing significant differences in KM curves for 6 genes. Notably, genes *PER1* (*p* = 0.003), *LPL* (*p* = 0.003), *G0S2* (*p* = 0.006), and *TFP1* (*p* = 0.012) were identified as the most significant risk factors for gastric carcinoma survival, as higher expression levels of these genes were associated with poorer prognosis (Figure 1F–I).

These prognostic genes reflect diverse biological mechanisms implicated in gastric tumorigenesis. For example, *PER1*, which is a circadian regulator, has been associated with advanced disease stages in gastric cancer [25], while *LPL* is linked to lipid metabolism and metastasis [26, 27]. The inclusion of both immune‐related (e.g., *G0S2*) and metabolic (e.g., *FABP3*, *LPL*) genes underscores the multifactorial nature of Hp‐driven gastric cancer progression. Their consistent association with patient outcomes supports their value as prognostic biomarkers.

### 3.3 Robustness of Prognostic Model

**FIGURE 2:**
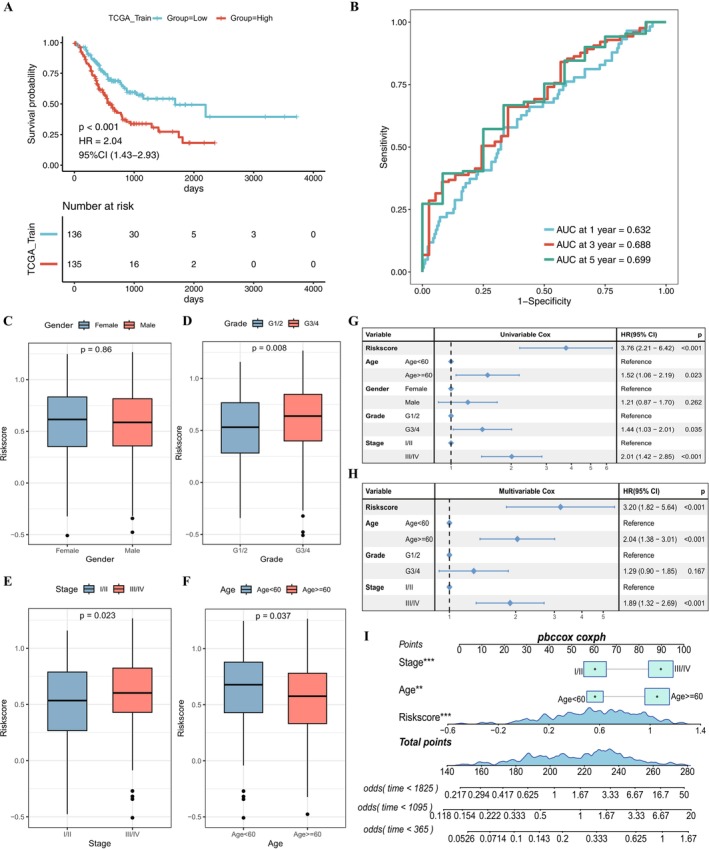
Evaluation of model prognostic performance in TCGA training set and independence test set. (A) Kaplan–Meier curve for TCGA training set. The *p* values were calculated from log‐rank test. (B) ROC curve for predication at 1, 3 and 5 years. (C–F) Box plots showing the distribution difference of risk scores among different clinical feature groups including gender, tumor grade, tumor stage and age. The *p* values were calculated from Wilcox rank test. (G, H) Forest plots showing the results of univariable and multivariable Cox analyses for clinical features in the TCGA cohort. (I) Nomogram plot of the prediction model. Square with attached line segments represents the contribution of each clinical factor to the outcome event. “Total Points” indicates the total score obtained by adding up the single scores corresponding to all variable values, and the three lines at the bottom represent the survival probabilities at 1, 3 and 5 years for each value point.

To evaluate the prognostic efficiency of the 9 risk signatures, a risk score model was constructed using Lasso regression coefficients with the STAD training set. The risk score was modeled as Risk Score = C6*0.043 + G0S2*0.01 + CTSK*0.065 + GLDN*0.116 + FABP3*0.008 + PER1*0.016 + LPL*0.00038 + TFPI*0.057 + CP*0.018. Each patient's risk score was calculated, and the training set was divided into high‐risk and low‐risk groups using the median risk score (Table S3). High‐risk patients showed higher expression of these signatures, shorter survival times, and more deaths (Figure S2H,I). KM curves indicated significantly poorer prognosis for the high‐risk group (*p* < 0.001) (Figure 2A). ROC curves showed AUC values of 0.632, 0.688 and 0.699 at 1, 3 and 5 years, respectively (Figure 2B).

**FIGURE 3:**
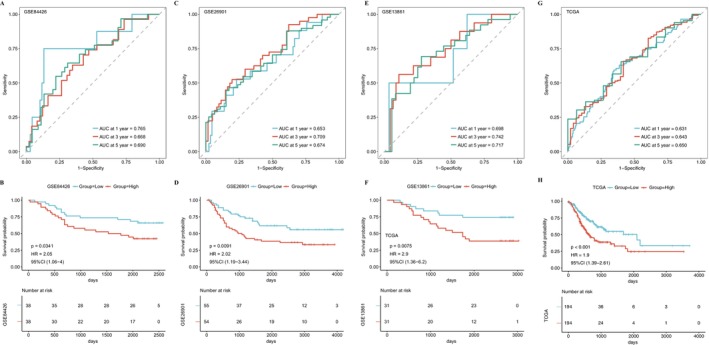
Validation of model prognostic performance. Kaplan–Meier curves and ROC curves for predication at 1, 3 and 5 years between risk score groups for GSE84426 (A, B), GSE26901 (C, D), GSE13861 (E, F) and overall TCGA set (G, H), respectively. Red represents high‐risk group and blue represents low‐risk group. Scatter plot of risk scores *p* for Kaplan–Meier curve was calculated from log‐rank test.

To validate the model, three external datasets (GSE84426, GSE26901, GSE13861) were used, which showed similar patterns and AUC values above 0.65 at 1, 3, and 5 years, confirming the model's efficacy and robustness in predicting gastric cancer prognosis (Figure 3A–F). A similar method was applied to the entire STAD dataset, yielding consistent results. High‐risk patients had poorer prognosis, and AUC values at 1, 3, and 5 years remained stable (Figure 3G,H). The reproducibility of the risk model across multiple independent cohorts highlights its translational potential. Our validation suggests that Hp‐associated signatures may represent fundamental biological processes in gastric cancer.

### 3.4 Evaluation of Risk Score Model

To explore the distribution of samples with different risk scores across various clinical characteristics, STAD patient data were grouped based on different clinical information including gender (Female vs. Male), tumor grades (G1/2 vs. G3/4), stage (Stage I/II vs. Stage III/IV) and age (< 60 years vs. ≥ 60 years). Expect for gender, significant differences for tumor grades (*p* = 0.008), stages (*p* = 0.023) and age (*p* = 0.037) were observed between the two risk groups (Figure 2C–F).

To verify whether the risk score can serve as an independent prognostic factor alongside other clinical features, univariate Cox regression analyses were conducted for risk score, age, gender, grade, and stage of gastric cancer (Table S3), followed by multivariate Cox regression including all these features together. When considered independently, features including risk score, age, stage, and grade were significant risk factors in gastric cancer (Figure 2G). In the multivariate Cox model, risk score, age, and stage remained significant risk factors after adjusting for other factors (Figure 2H), indicating their independent status as prognostic factors. Lastly, based on the survival outcomes of the cohort, nomograms were constructed for the independent prognostic factors, identifying risk score and tumor stage as the two most significant contributors for gastric cancer prognosis evaluation (Figure 2I). Overall, the ability of the risk score to predict outcomes independently of clinical features such as age and tumor stage suggests that it captures underlying biological features of disease aggressiveness. This complements current staging systems and could help identify high‐risk patients who may benefit from intensified treatment strategies.

### 3.5 Differential Pathway Enrichment Between Groups

**FIGURE 4:**
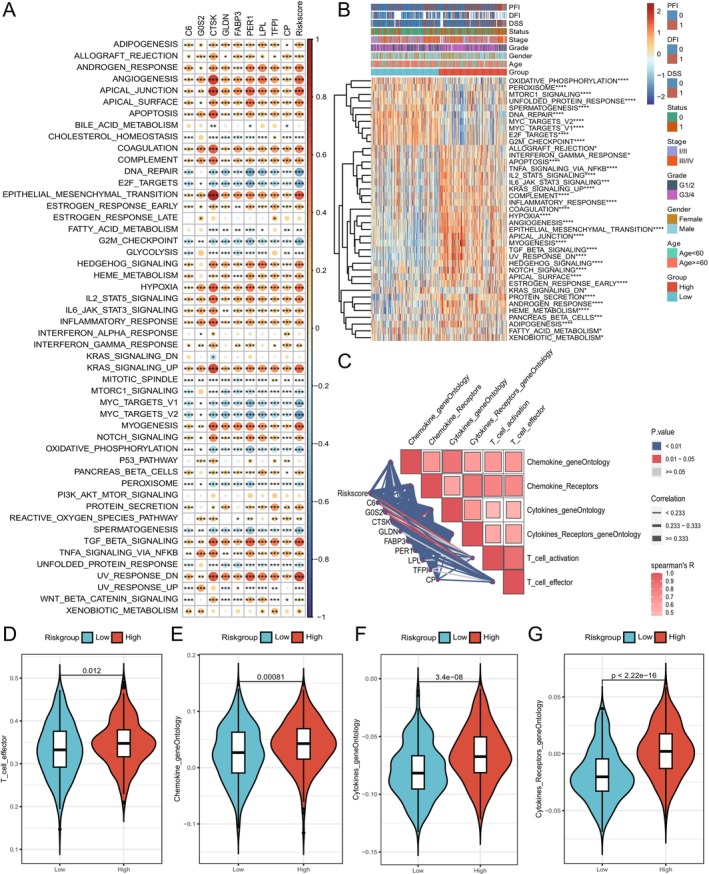
Differences in gene set enrichment between risk groups. (A) Heatmap showing the correlation between risk score, expression of model genes, and pathway enrichment scores. Color intensity indicating the strength of correlation and “*” indicating significance. (B) Differences in HALLMARK pathway enrichment scores between high and low‐risk groups, with “*” indicating significance. (C) Plot showing the correlation between risk score, expression of model genes, and enrichment scores of immune function gene sets. (D–G) Violin plots showing the distribution differences of enrichment scores for four immune function gene sets between high and low‐risk groups.

Based on the established risk score model, we explored the functional pathways associated with the prognostic signatures and the different risk groups to understand the relationship between cancer characteristic pathways and the prognostic model. First, ssGSEA was performed for the prognostic signatures by calculating their correlation with HALLMARK pathways as enriched scores, followed by a heatmap comparing the enriched scores between high and low‐risk groups and other clinical features such as age, gender, grade, and stage (Figure 4A,B, Table S4). Consistently, positively enriched pathways showed higher enrichment scores in the high‐risk group, such as ANGIOGENESIS, APOPTOSIS, COAGULATION, EPITHELIAL_MESENCHYMAL_TRANSITION, INFLAMMATORY_RESPONSE, and TGF_BETA_SIGNALING, while negatively associated pathways had lower enriched scores in the high‐risk group, such as DNA_REPAIR, G3M_CHECKPOINT, MYC_TARGET_V1/2, and SPERMATOGENESIS. These findings align with the notion that high‐risk tumors are characterized by enhanced angiogenesis, inflammation and epithelial–mesenchymal transition, which are hallmarks of tumor invasiveness [28]. Conversely, reduced enrichment in DNA repair and cell cycle checkpoint pathways may reflect genomic instability in aggressive tumors, consistent with previous reports in gastric cancer [29].

Next, the association between the risk score model and immune functions was assessed by calculating the correlation between risk score, gene expression of signatures, and the enrichment scores of immune function gene sets. All model signatures and risk score were positively associated with cytokines, cytokine receptors, chemokines, and T cell activation (Figure 4C). Furthermore, the high‐risk group had significantly higher enrichment scores compared to the low‐risk group across the four immune gene sets, indicating distinct immune activities in the two risk groups (Figure 4D–G).

### 3.6 Differential Tumor Immune Microenvironment Between Groups

**FIGURE 5:**
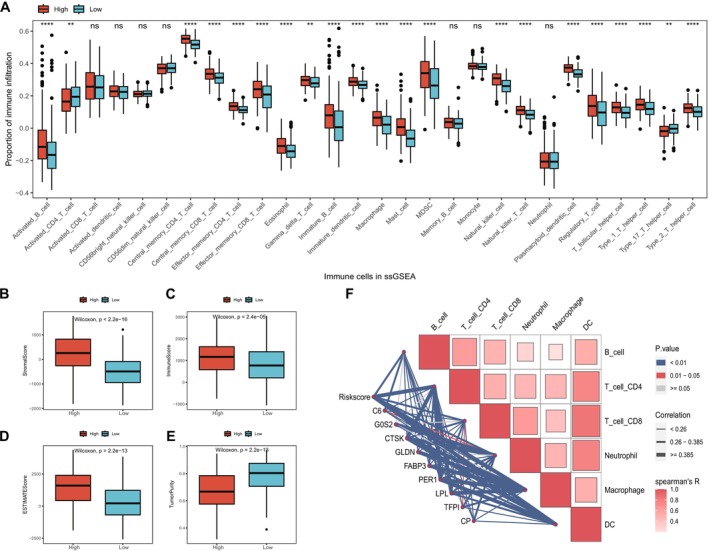
Relationship between risk model and tumor immune microenvironment. (A) Boxplot showing the differences in immune cell infiltration proportions between high and low‐risk groups generated from the ssGSEA algorithm. Red represents the high‐risk group, blue representing the low‐risk group, and “*” indicating difference significance. (B–E) Boxplots showing the differences in stromal score, immune score, ESTIMATE score, and tumor purity between high and low‐risk groups. (F) Correlation between the infiltration proportions of six immune cell types calculated by the TIMER algorithm and the risk score and expression of model genes.

To investigate the differences in tumor immune microenvironment between high and low‐risk groups, we initially analyzed the expression of immune regulatory factors between these groups. Generally, immune regulatory factors exhibited higher expression levels in the high‐risk group (Figure S3A and Table S5). The immune cell infiltration proportions were estimated and compared for 28 immune cell types between the two groups (Figure 5A and Table S6). Among them, 21 immune cell types showed significant differences.

We then compared the four index scores calculated by ESTIMATE including immune scores, tumor purity, stromal scores, and ESTIMATE scores between two risk groups. Among them, stromal score, immune score, and ESTIMATE score were significantly higher in the high‐risk group, while tumor purity was lower compared to the low‐risk group (Figure 5B–E). Furthermore, the correlation between the infiltration proportions of six immune cell types calculated by the TIMER algorithm and the risk score and signature gene expression was shown in Figure 5F. Macrophages exhibited the strongest correlation with risk score and signature expression. The observed immune cell infiltration patterns reinforce the central role of the tumor immune microenvironment in Hp‐driven gastric cancer. Elevated stromal and immune scores in high‐risk patients suggested an immunosuppressive milieu, particularly driven by macrophages, which have been widely implicated in promoting tumor progression and immune evasion [30]. These results further support the involvement of Hp‐related immune dysregulation in shaping tumor behavior.

### 3.7 Differences in Genomic Mutations Between Risk Groups

**FIGURE 6:**
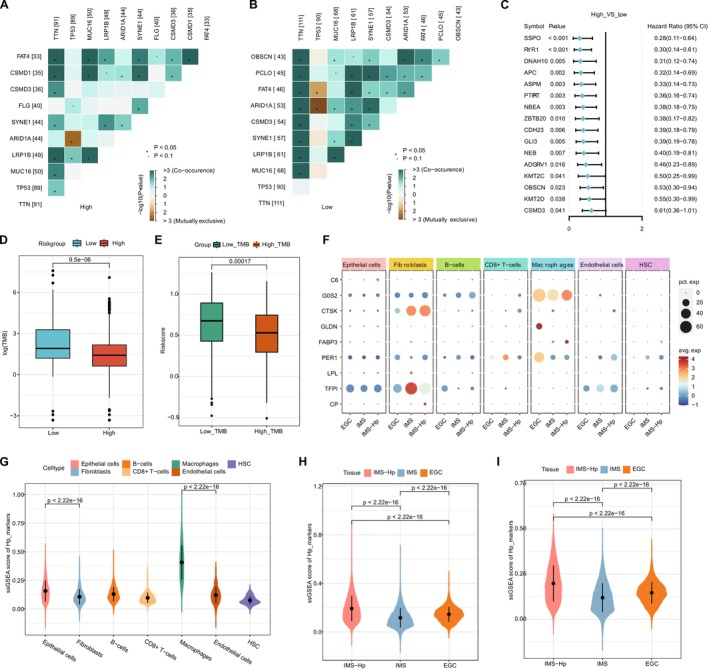
Mutation analysis between risk groups and single‐cell analysis. (A, B) Heatmaps showing the co‐occurrence and mutual exclusivity of the top 10 genes with the highest mutation frequency in the high and low‐risk groups. (C) Mutation differences in the top 100 mutated genes of the TCGA‐STAD cohort between the high and low‐risk groups. (D) Differences in Tumor Mutation Burden (TMB) between high and low‐risk groups. (E) Differences in risk scores between high and low TMB groups. (F) Expression of model genes in different cell types across various tissues. Size of the bubbles represents the number of cells expressing the gene, and the color indicates the average expression level. (H, I) Enrichment differences of marker genes related to Hp infection in different cell types, tissue sources, and epithelial cells within different tissues.

Given the significant role of gene mutations in cancer progression, studying mutations at the genomic level is crucial for the development of targeted therapies and novel cancer treatments. We evaluated mutation profiles between the two STAD risk groups. Firstly, to demonstrate the distribution of somatic variations among samples between high and low‐risk groups and to illustrate the distribution of gene mutations among samples with different clinical characteristics, the profiles of the top 30 genes with the highest mutation frequency in high and low‐risk groups were presented in Figure S3B,C. Genes *TTN*, *TP53*, and *MUC16* had the highest mutation frequencies, with TTN and MUC16 more frequent in the low‐risk group. We analyzed mutation patterns of the top 10 highly mutated genes from each group, finding stronger co‐occurrence and mutual exclusivity in the low‐risk group (Figure 6A,B).

Among the top 100 mutated genes, 16 showed significant differences between risk groups (Figure 6C). Tumor Mutation Burden (TMB) was higher in the low‐risk group (Figure 6D). Using the median TMB value, significant differences in risk scores were observed between high and low TMB groups (Figure 6E). These results highlight the mutation differences between the two risk groups. The higher TMB observed in low‐risk patients suggested elevated mutational load may enhance tumor immunogenicity and responsiveness to immunotherapy. In contrast, high‐risk patients, despite having poorer outcomes, showed lower TMB, which could explain their reduced sensitivity to immune checkpoint inhibitors. These genomic differences highlight the importance of integrating mutational features with transcriptomic risk scores in guiding treatment decisions.

### 3.8 Predicting Treatment Efficacy With Risk Models

To assess the immune treatment response of patients from the two risk groups, we extracted clinical treatment data from the immunotherapy IMvigor210 cohort. IMvigor210 is a multicenter, single‐arm, 2‐cohort phase 2 trial investigating the efficacy and safety of atezolizumab in metastatic urothelial cancer. Initially, the risk scores of samples in the cohort were calculated (Table S7). Patients showing partial or complete response to immune therapy had lower risk scores (Figure S4A). The IMvigor210 cohort patients were divided into high and low‐risk groups based on the median value of risk scores, presenting different distributions of responders and non‐responders (Figure S4B). These results suggest the prognostic capability of our risk score model in predicting immune therapy efficacy.

To predict the immune response status of STAD patients, we employed TIDE (Tumor Immune Dysfunction and Exclusion) to evaluate the potential of tumor immune escape based on gene expression profiles (Table S7). Patients from the STAD cohort were compared based on their predicted status and risk scores. Consistently, patients predicted with responses showed lower risk scores, and more responders were found in the group with lower risk scores (Figure S4C,D).

Based on the predictive results from TIDE, correlations between risk score and TIDE score, T cell exclusion score, T cell dysfunction score, infiltration of cancer‐associated fibroblasts (CAFs) and myeloid‐derived suppressor cells (MDSCs) were evaluated (Figure S4E–H). Results showed that risk scores were significantly positively correlated with TIDE scores, T cell dysfunction scores, and CAFs, while negatively correlated with infiltration of MDSCs. Moreover, since Immunophenoscore (IPS) can determine tumor immunogenicity and predict response to immunotherapy, we analyzed differences in immune response between the two risk groups. We compared IPS scores of four categories (ips_ctla4_neg_pd1_neg, ips_ctla4_pos_pd1_neg, ips_ctla4_neg_pd1_pos, ips_ctla4_pos_pd1_pos) between high and low‐risk groups (Table S7). Results in Figure S4I–L showed that IPS scores of all four categories were significantly higher in the low‐risk group compared to the high‐risk group, indicating that patients in the low‐risk group were more likely to benefit from immunotherapy. These observations suggested that our risk score not only predicts prognosis but also stratifies patients for immunotherapy responsiveness. Particularly, low‐risk patients with higher IPS scores appear more immunogenic and likely to benefit from checkpoint blockade.

### 3.9 Expression Distribution of Prognostic Factors in scRNA‐Seq Data

To explore the cell type‐specific expression profile of prognostic factors in the risk model, we analyzed the expression characteristics of prognostic genes at the single‐cell level. A total of seven major cell types (epithelial cells, fibroblasts, macrophages, endothelial cells, B cells, CD8+ T cells, and HSC cells) were identified from gastric cancer samples (Figure S5A). By comparing cell distribution among early gastric cancer tissue, intestinal metaplasia tissue, and Hp‐infected intestinal metaplasia tissue, we found that macrophages showed a stronger increase in Hp‐infected tissue (Figure S5B). The expression levels of genes in the risk model were also compared among the three tissues, and they were highly expressed in macrophages and fibroblasts. Particularly, *G0S2* and *CTSK* were highly expressed in Hp‐infected macrophages and fibroblasts, while *TFPI* was expressed lower in Hp‐infected fibroblasts and *GLDN* showed higher expression in macrophages of early gastric cancer (Figure 6F). Moreover, *CTSK* and *TFPI* served as marker genes for fibroblasts, and *G0S2* was a marker for macrophages (Figure S5C).

By performing ssGSEA, the enrichment scores for associated gene sets with each risk factor were calculated and compared across cell types and tissues. Higher enrichment scores were observed for macrophages and gastric cancer tissue (Figure 6G,H). For epithelial cells, enrichment scores of factors in gastric cancer were higher than those in intestinal metaplasia (Figure 6I). These results indicated the association between Hp‐related risk factors with different cell types in gastric tissues. The enrichment of key prognostic factors in fibroblasts and macrophages highlights their pivotal role in the Hp‐infected gastric microenvironment. Particularly, macrophages are known to facilitate tumor‐promoting inflammation and immune suppression [30], while fibroblasts contribute to stromal remodeling and invasion. The single‐cell analysis therefore provides mechanistic insight into how Hp‐related transcriptional alterations converge on specific cellular compartments.

### 3.10 Cell Types Expressing Key Prognostic Factors

To further illustrate the molecular features for different cell types from gastric cancer data, we analyzed the inflammatory response and cytokine enrichment levels in various cell groups. As shown in Figure S6A,B, fibroblasts and macrophages exhibited the highest levels of inflammatory response and cytokine enrichment. Moreover, marker genes from fibroblasts were enriched in functional pathways such as cancer‐associated glycosaminoglycans, antigen processing and presentation, viral infection, inflammatory response, atherosclerosis, and diabetes, while markers from macrophages were mainly enriched in pathways such as autoimmune diseases, antigen processing and presentation, *Staphylococcus aureus* infection, type I diabetes, intestinal immune network for *IgA* production, transplant rejection, assembly and processing of MHC class II‐associated proteins, antigen processing, and presentation of exogenous antigens (Figure S6C–F). These pathways, particularly those from macrophages, largely overlapped with those from commonly identified dysregulated genes in gastric carcinoma related to Hp infection, indicating the association between Hp infection‐related genes and fibroblasts and macrophages. Specifically for macrophages, we examined the expression of genes *G0S2* and *CTSK* at the pan‐cancer single‐cell level, and *G0S2* was significantly higher in macrophages across most cancer datasets, while *CTSK* was significantly higher in fibroblasts across multiple cancers (Figure S6G,H). Altogether, these results indicated the potential functional relationship of Hp infection‐related genes with fibroblasts and macrophages in gastric cancer.

## 4 Discussion

In this study, we developed a risk model for gastric cancer by identifying prognostic factors associated with Hp infection across multiple public datasets. Initially, we conducted a comprehensive analysis of differential gene expression in three GEO datasets containing Hp‐infected gastric tissue, discerning genes that differed between Hp‐infected and uninfected tissue. Subsequently, by intersecting the differential results from these datasets, we identified a set of common differential genes. Utilizing single‐factor Cox analysis in the TCGA‐STAD cohort, we further identified genes significantly associated with gastric cancer prognosis. Finally, we constructed a predictive risk scoring model for gastric cancer prognosis, comprising nine genes related to differences in Hp infection.

Some of the prognostic factors identified in our study have been previously associated with gastric cancer. For example, *CTSK* (Cathepsin‐K) has been implicated in tumor invasion and metastasis in various cancers, including gastric cancer [31, 32, 33]. Particularly, Chang et al. [32] found that the expression level of *CTSK* and immunological checkpoints were positively associated, which indicated its potential implication to improve the immune efficacy of gastric cancer. Additionally, *TFPI* (Tissue Factor Pathway Inhibitor) is a protease inhibitor that regulates the initiation of blood coagulation by inhibiting the tissue factor pathway [33]. Ceruloplasmin, known for its role in iron metabolism, has been found upregulated in various cancers, including lung adenocarcinoma cells, premalignancies, oral cancer, and clear‐cell renal cell carcinoma, although its specific functions in gastric tumors require further investigation [34, 35, 36]. One clinical trial study reported that ceruloplasmin, along with adjuvant polychemotherapy, in 30 patients with gastric cancer found that normalization of the levels of natural ceruloplasmin and iron in the blood serum occurred in patients [37].

*PER1* (Period Circadian Regulator 1) regulates the circadian rhythm, participating in the negative feedback loop of the circadian clock by inhibiting the activity of *CLOCK‐BMAL1* transcriptional complexes. Deregulation of *PER1* expression has been found to be associated with tumor development and progression in cancers such as breast cancer, gliomas, and gastric cancer [25, 38, 39]. Zou et al. [25] found that low levels of *PER1* expression were more frequently observed in gastric cancer patients with depth of invasion or those at advanced stages. This was consistent with our results that included *PER1* as a risk factor in gastric cancer. Similarly, *G0S2* (G0/G1 switch gene 2) was originally identified as being transiently induced in human peripheral blood mononuclear cells during the transition from G0 to G1 phase [25]. *G0S2* has been reported to have antitumor activity as it was found to be epigenetically silenced by gene promoter methylation in adrenocortical carcinomas, lung cancer, and various cancer cell lines [25, 36, 37, 38, 39, 40, 41, 42, 43, 44]. *FABP3* is a member of the fatty acid binding protein family, involved in the intracellular transport and metabolism of fatty acids. It facilitates the uptake and trafficking of fatty acids within cells. Higher expression levels of *FABP3* have been associated with increased depth of vascular invasion and metastases stage in gastric adenocarcinomas. Its role in lipid metabolism and energy homeostasis may influence the aggressiveness of gastric tumors [45]. Lipoprotein lipase (*LPL*) is an important gene in lipid metabolism, particularly engaging in the breakdown of extracellular fat to create exogenous fatty acids, playing a crucial role in the uptake of lipids by peripheral tissues for energy production or storage. It has also been reported to promote gastric cancer metastasis, which indicated a poor prognosis of gastric cancer [26, 27].

The prognostic genes identified in our study were validated through multiple approaches. We assessed the prediction efficacy of the risk model using the TCGA training set, the overall set, and three external validation sets from GEO, confirming its stable prediction results. Moreover, results from single and multiple‐factor Cox analyses indicated that the risk score can serve as an independent prognostic factor. Comprehensive evaluations illustrated significant associations between the risk score and clinical features, HALLMARK functional enrichment, immune responses, genomic mutations, and expression of immune modulators in gastric cancer. All these results illustrated that patients with higher risk scores tend to be at a more severe stage of tumor development.

Furthermore, our analysis of immune therapy response revealed significant differences between high and low‐risk groups, suggesting that model genes may serve as markers for immune response factors in gastric cancer. Lastly, through single‐cell data analysis, we examined the expression distribution of model genes across cell types, highlighting their potential effects on macrophages. Tumor‐associated macrophages play crucial roles in the tumor microenvironment, supporting cancer progression through various mechanisms [30].

## 5 Conclusion

In summary, our study provides a comprehensive evaluation of the prognostic model and solid evidence supporting its robustness and prognostic effect in gastric cancer. However, further in vivo and in vitro validation is necessary to validate the findings of this study.

## Author Contributions

Zhiying Tian was responsible for the study design and manuscript writing. Li Zhang conducted the literature review. Miao Su performed the statistical analysis. Bin Yang collected the data. Zhaoyun Zhang contributed to manuscript editing and proofreading.

## Funding

The research was supported by the Hengshui Science and Technology Bureau under the science and technology project titled “Study on the Correlation between *Helicobacter pylori* Typing Combined with Gastric Function Detection and Benign and Malignant Diseases of the Stomach and Duodenum,” Project Number: 2020014049Z.

## Ethics Statement

The data used in this study were all publicly available. Human and animal studies were not conducted. So, human and animal ethics are not applicable.

## Consent

The authors have nothing to report.

## Conflicts of Interest

The authors declare no conflicts of interest.

## Supporting information


**Figure S1:** A workflow illustrating the study design and major outcomes.

**Figure S2:** DEGs between Hp‐infected and uninfected tissues.

**Figure S3:** Tumor immune microenvironment and mutation profiles between risk groups.

**Figure S4:** Prediction of immunotherapy efficacy by the risk model.

**Figure S5:** Heterogeneity of gastric cancer in single‐cell data.

**Figure S6:** Expression profiles of key prognostic factors across cell types and pan‐cancer.

**Table S1:** Common DEGs.

**Table S2:** KEGG and GO enrichment analysis results for Common‐DEGs.

**Table S3:** Construction and verification results of HPRS.

**Table S4:** Enrichment score analysis results of the risk score model.

**Table S5:** Results of immune‐related factors.

**Table S6:** Proportion of immune‐infiltrating cells in the TCGA‐STAD cohort.

**Table S7:** Results of immunotherapy response analysis among model groups.

## Data Availability

Publicly available datasets were analyzed in this study. They were extracted from Gene Expression Omnibus (GEO) (http://www.ncbi.nlm.nih.gov/geo/) and the Genomic Data Commons (GDC) portal (https://portal.gdc.cancer.gov/).
