# Supplementary material for: Construction of a Prognostic Risk Model for Helicobacter pylori Infection in Gastric Cancer Patients and Immunological Analysis
Source: Cancer Rep (Hoboken). 2026 Apr 2;9(4):e70511. doi: 10.1002/cnr2.70511 (PMC13045470; doi:10.1002/cnr2.70511)
Supplement: Supplementary file 1 — Figure S1: A workflow illustrating the study design and major outcomes. [file CNR2-9-e70511-s012.pdf]

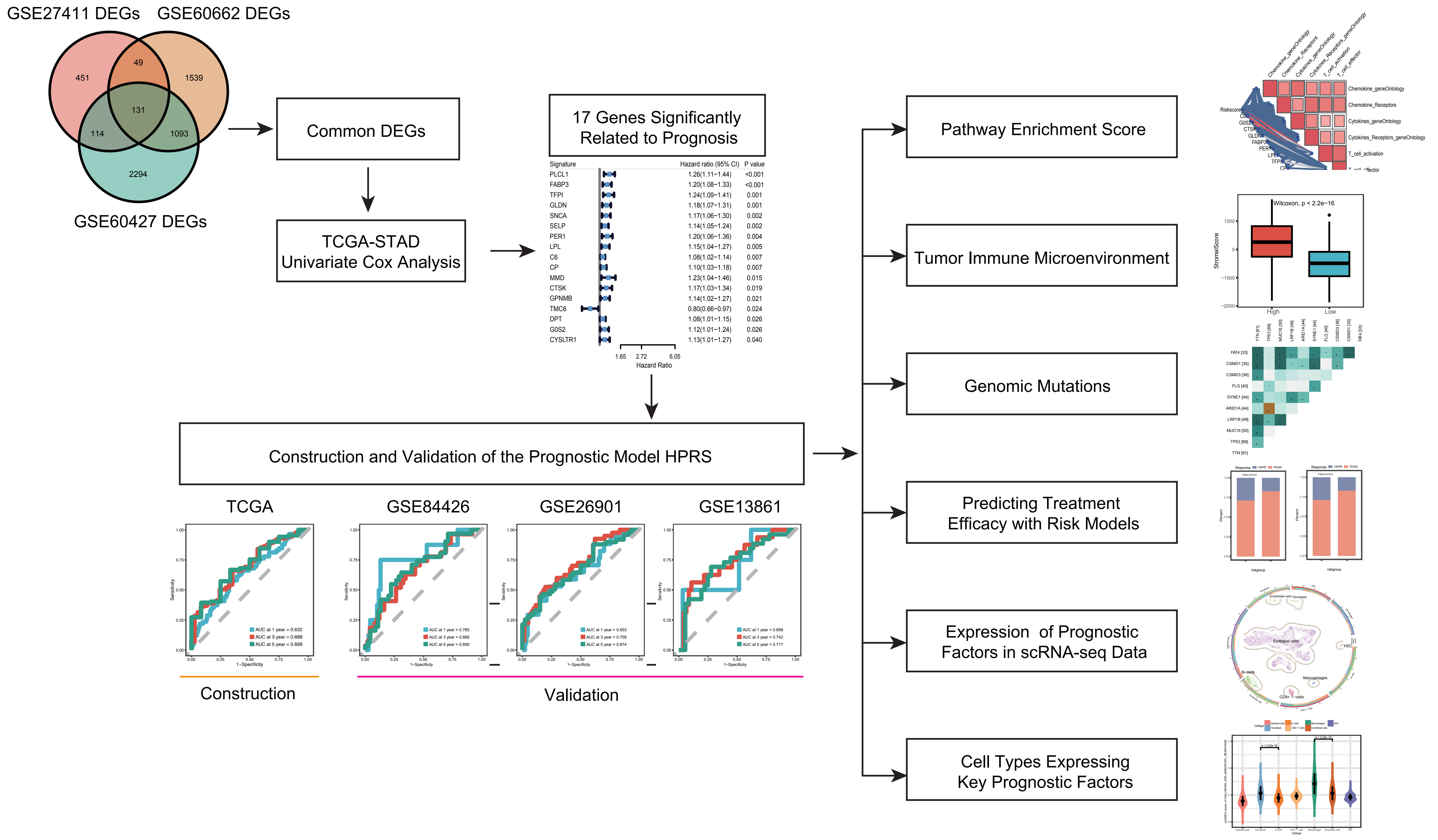
GSE27411 DEGs
GSE60662 DEGs
GSE60427 DEGs
451
49
1539
131
114
1093
2294
Common DEGs
TCGA-STAD
Univariate Cox Analysis
17 Genes Significantly
Related to Prognosis
Signature
Hazard ratio (95% CI)
P value
PLCL1 1.26(1.11−1.44) <0.001
FABP3 1.20(1.08−1.33) <0.001
TFPI 1.24(1.09−1.41) 0.001
GLDN 1.18(1.07−1.31) 0.001
SNCA 1.17(1.06−1.30) 0.002
SELP 1.14(1.05−1.24) 0.002
PER1 1.20(1.06−1.36) 0.004
LPL 1.15(1.04−1.27) 0.005
C6 1.08(1.02−1.14) 0.007
CP 1.10(1.03−1.18) 0.007
MMD 1.23(1.04−1.46) 0.015
CTSK 1.17(1.03−1.34) 0.019
GPNMB 1.14(1.02−1.27) 0.021
TMC6 0.80(0.66−0.97) 0.024
DPT 1.08(1.01−1.15) 0.026
G0S2 1.12(1.01−1.24) 0.026
CYSLTR1 1.13(1.01−1.27) 0.040
1.65
2.72
6.05
Hazard Ratio
Construction and Validation of the Prognostic Model HPRS
TCGA
GSE84426
GSE26901
GSE13861
AUC at 1 year = 0.632
AUC at 3 year = 0.688
AUC at 5 year = 0.699
AUC at 1 year = 0.765
AUC at 3 year = 0.668
AUC at 5 year = 0.690
AUC at 1 year = 0.653
AUC at 3 year = 0.709
AUC at 5 year = 0.674
AUC at 1 year = 0.698
AUC at 3 year = 0.742
AUC at 5 year = 0.717
Sensitivity
1−Specificity
Construction
Validation
Pathway Enrichment Score
Tumor Immune Microenvironment
Genomic Mutations
Predicting Treatment
Efficacy with Risk Models
Expression of Prognostic
Factors in scRNA-seq Data
Cell Types Expressing
Key Prognostic Factors
Chemokine_geneOntology
Chemokine_Receptors
Cytokines_geneOntology
Cytokines_Receptors_geneOntology
T_cell_activation
Riskscore
Wilcoxon, p < 2.2e−16
StromalScore
High
Low
Epithelial cells
Fibroblasts
Endothelial cells
B−cells
Macrophages
CD8+ T−cells
HSC
riskgroup
Response
Percent
