# Supplementary figures and images for: Construction of a Prognostic Risk Model for Helicobacter pylori Infection in Gastric Cancer Patients and Immunological Analysis
Source: Cancer Rep (Hoboken). 2026 Apr 2;9(4):e70511. doi: 10.1002/cnr2.70511 (PMC13045470; doi:10.1002/cnr2.70511)

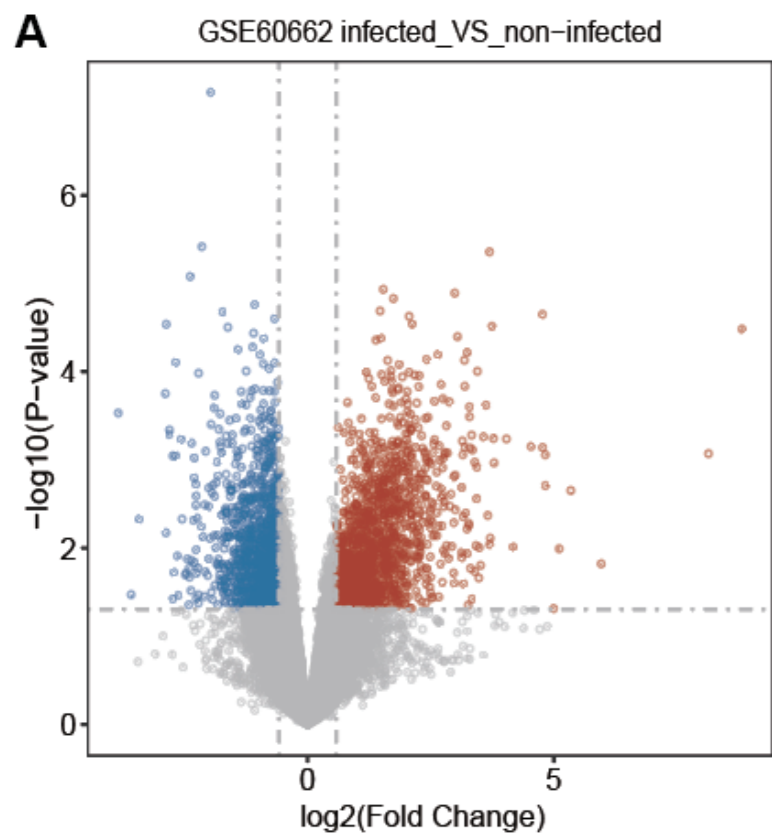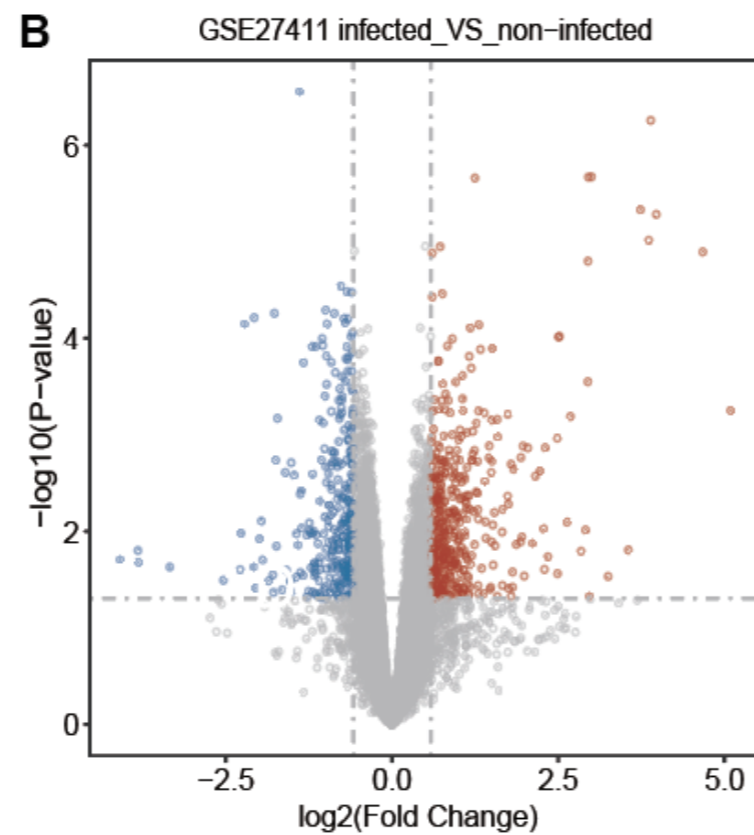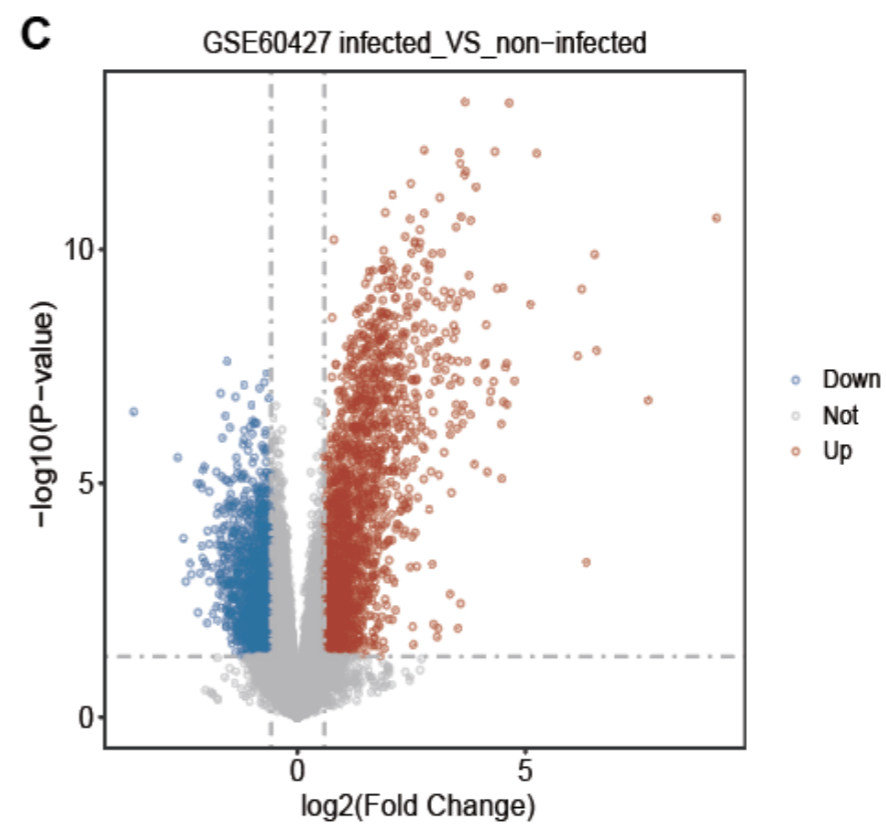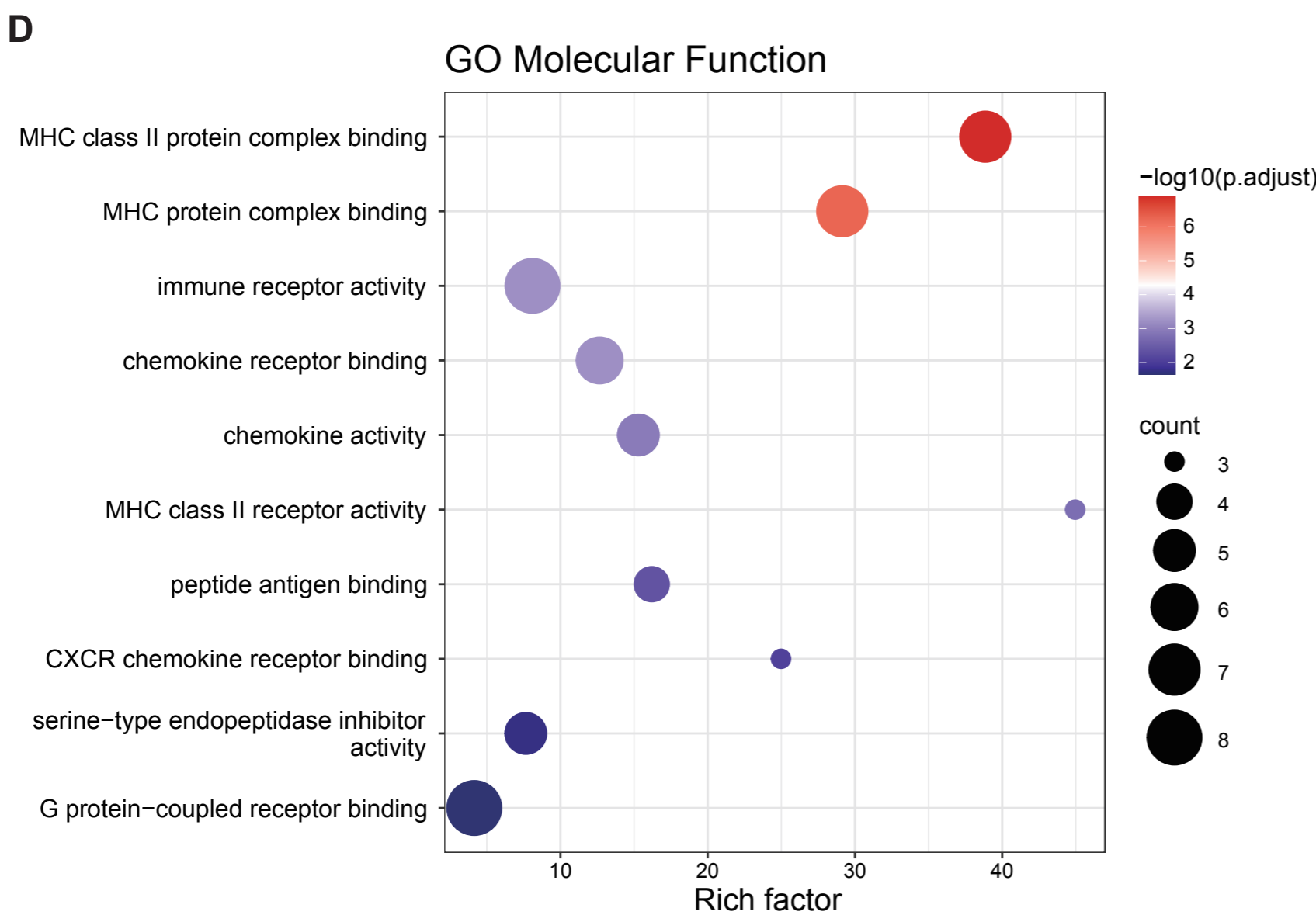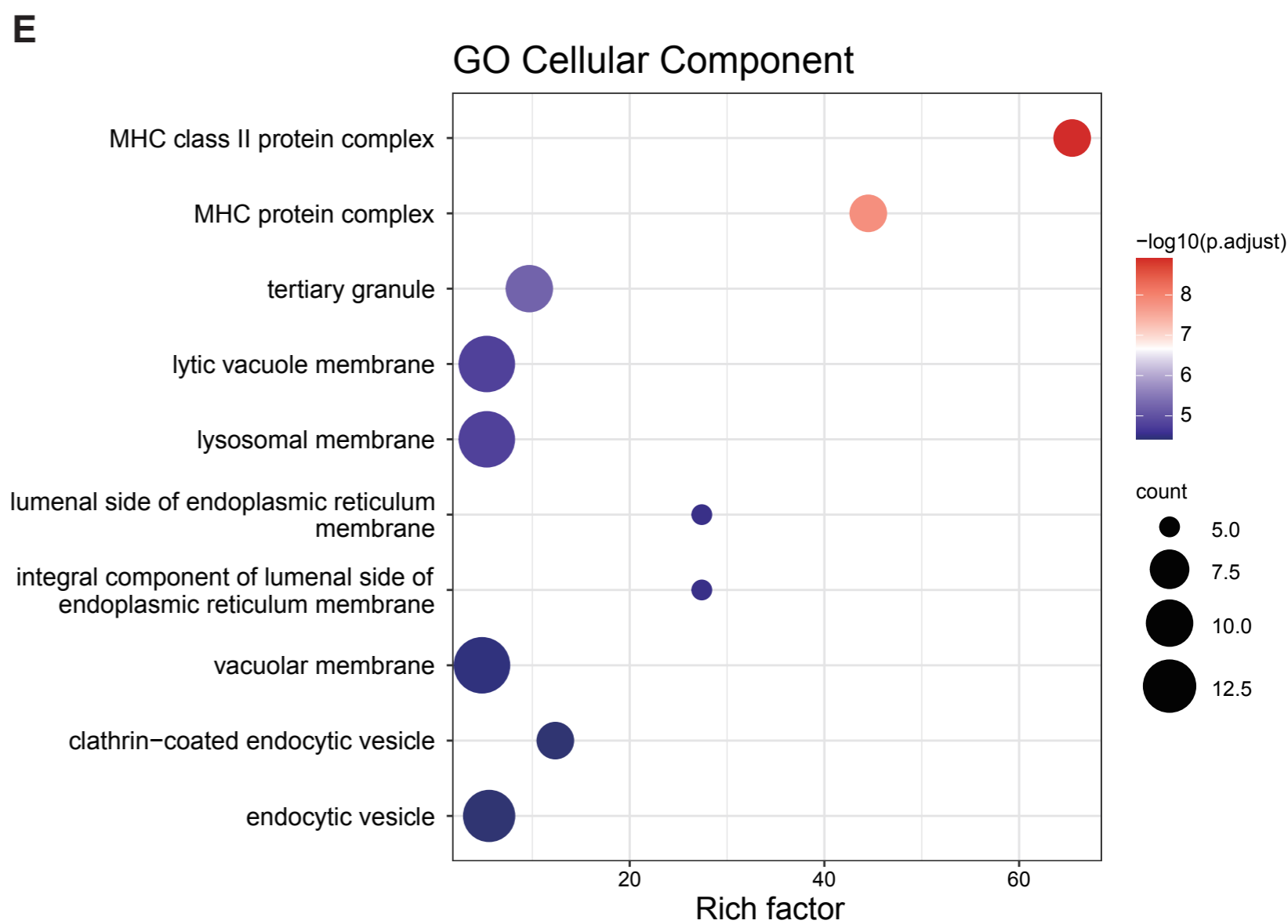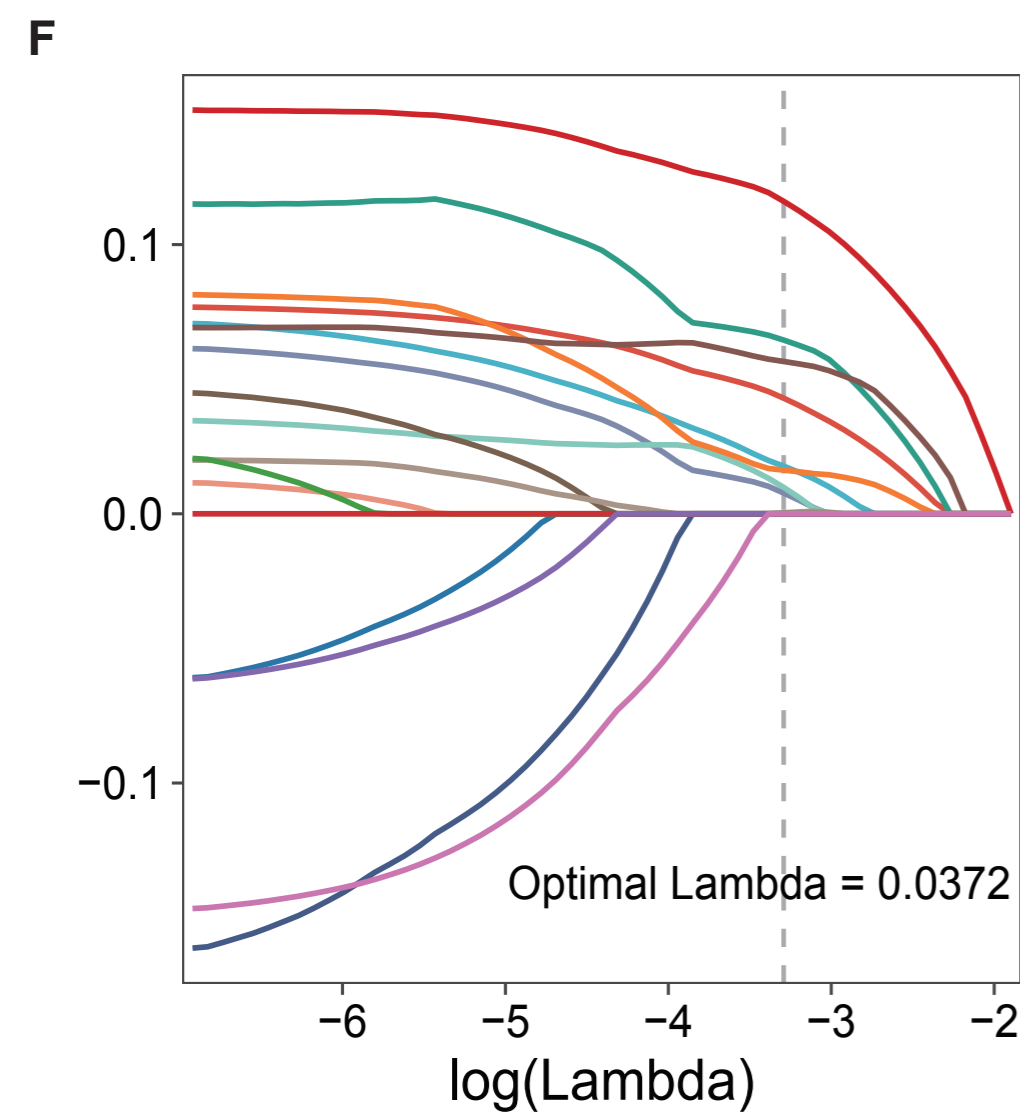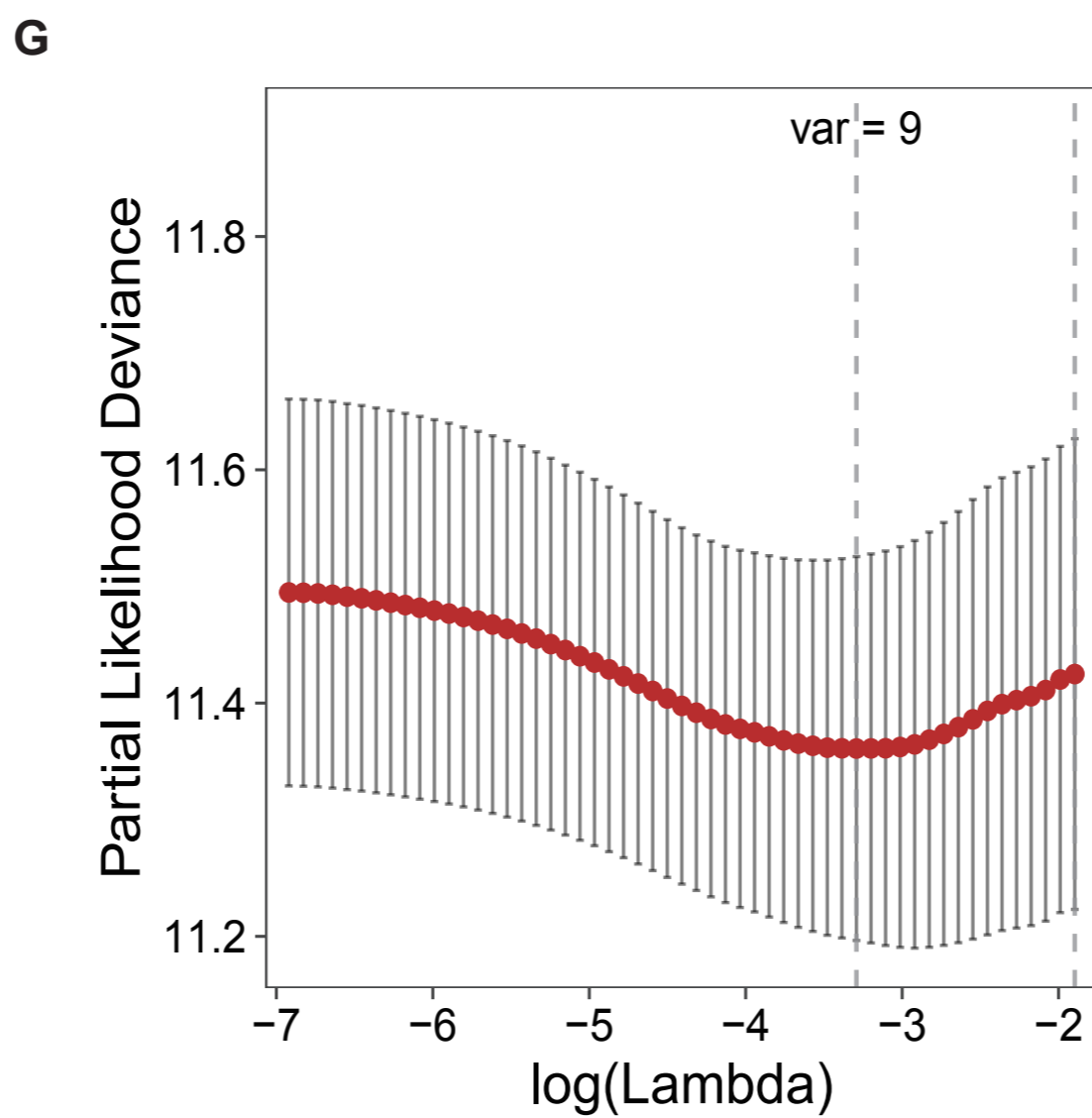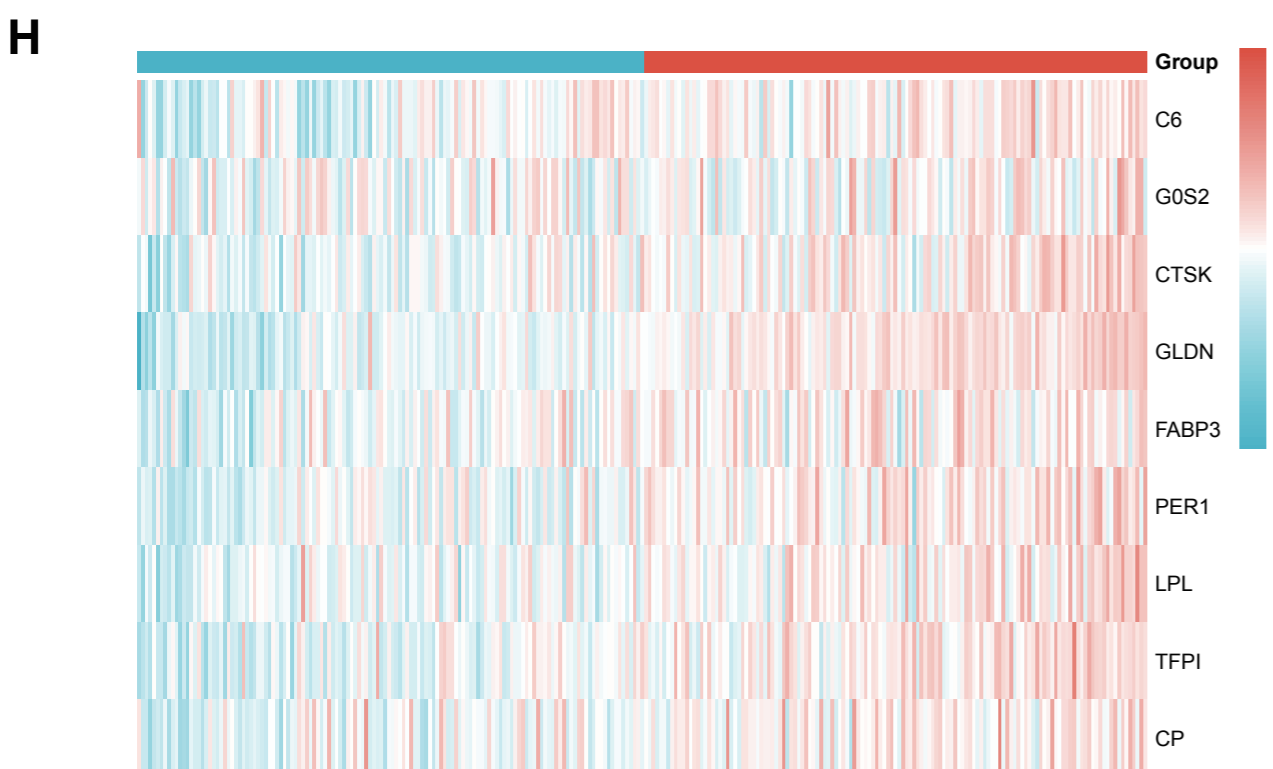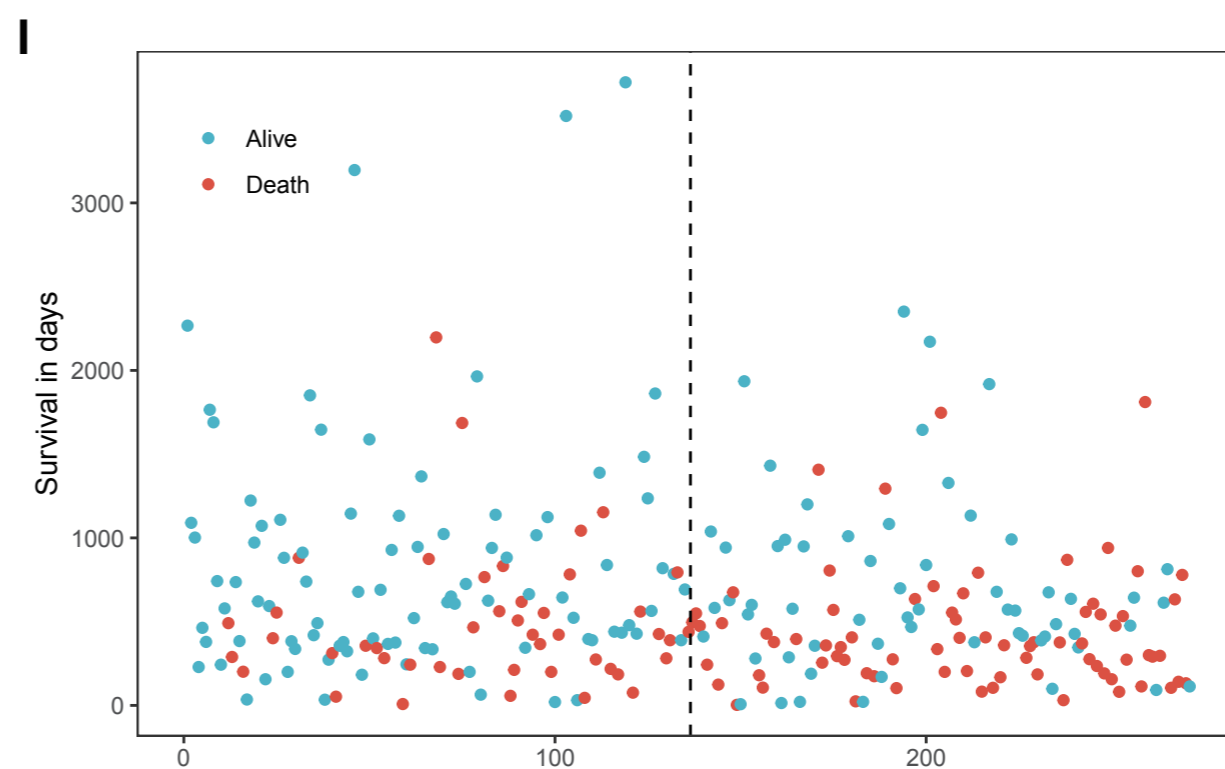

Supplement: Supplementary file 2 — Figure S2: DEGs between Hp‐infected and uninfected tissues. [file CNR2-9-e70511-s008.pdf]

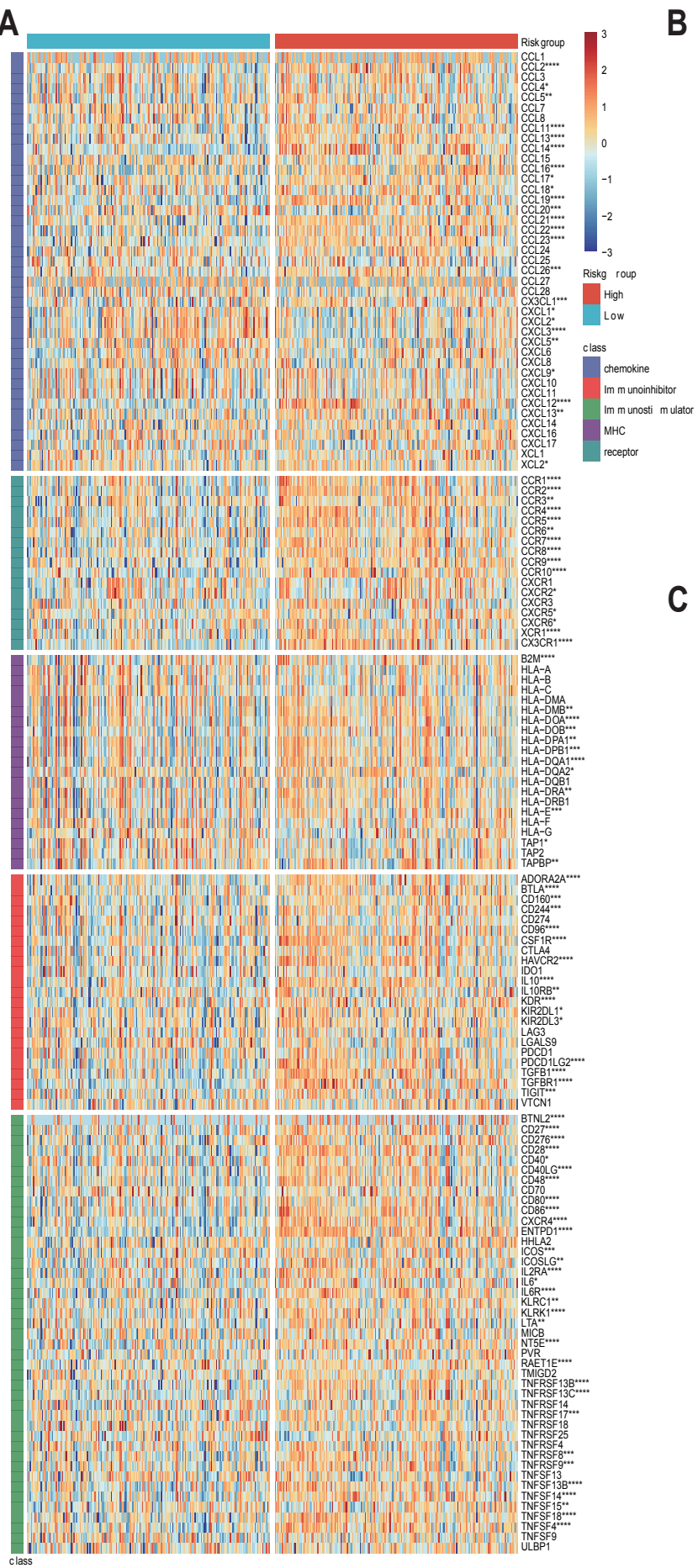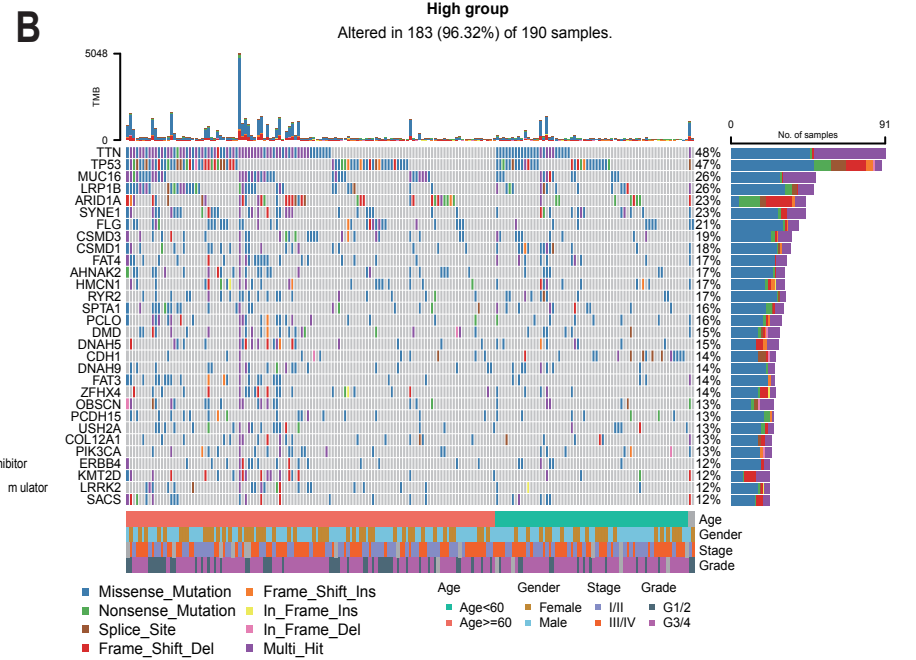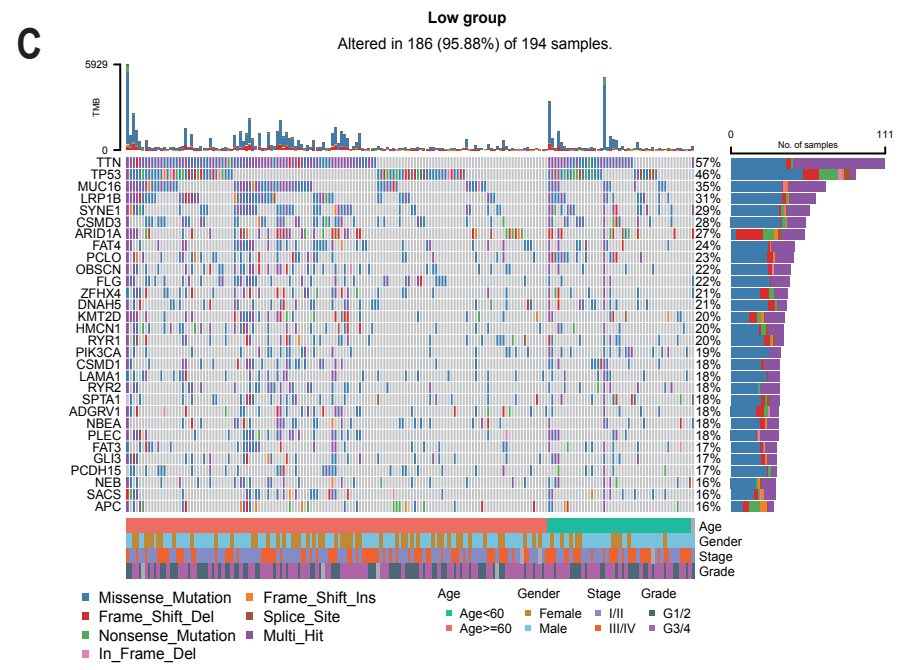

Supplement: Supplementary file 3 — Figure S3: Tumor immune microenvironment and mutation profiles between risk groups. [file CNR2-9-e70511-s002.pdf]

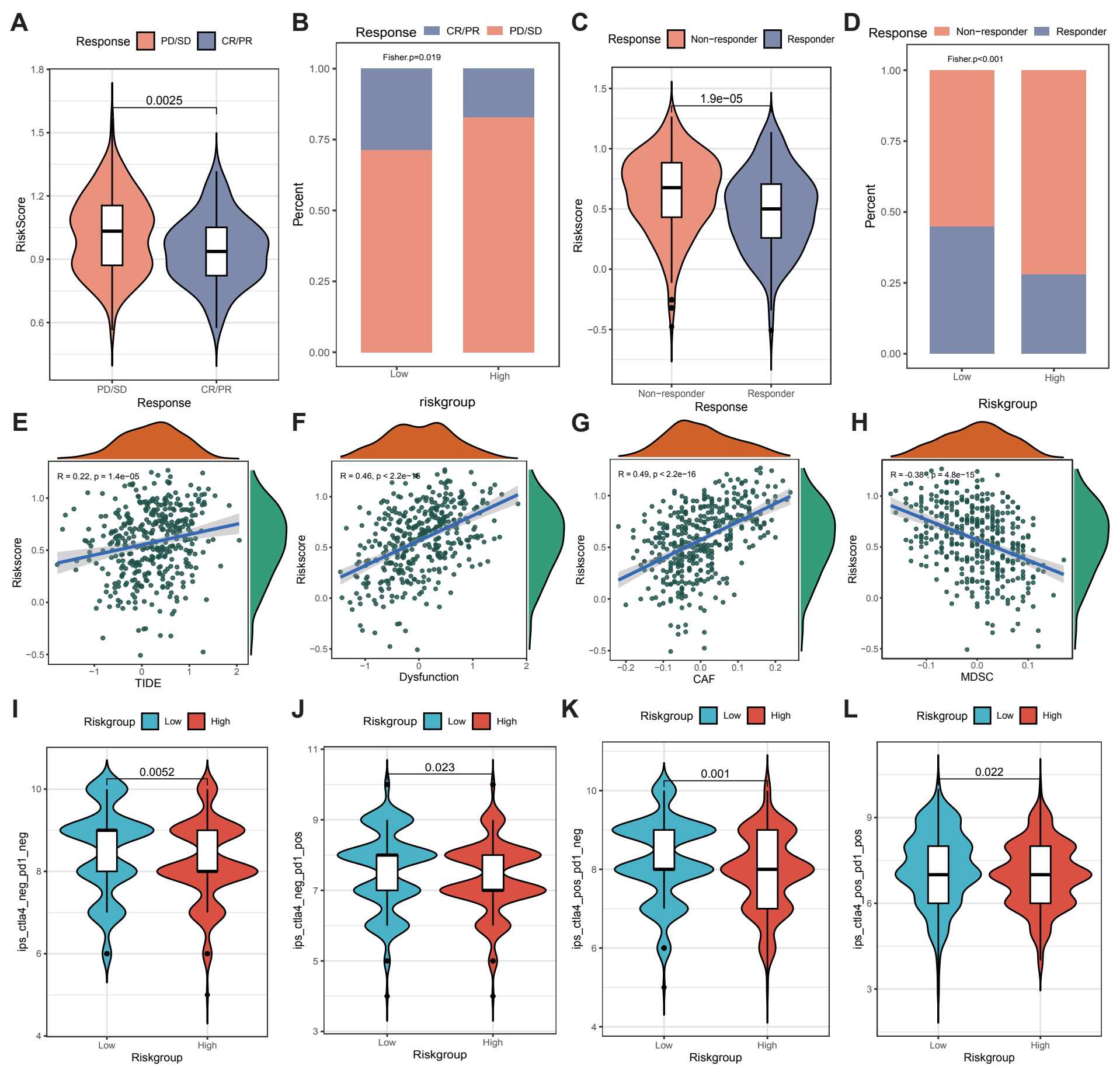

Supplement: Supplementary file 4 — Figure S4: Prediction of immunotherapy efficacy by the risk model. [file CNR2-9-e70511-s013.pdf]

**A**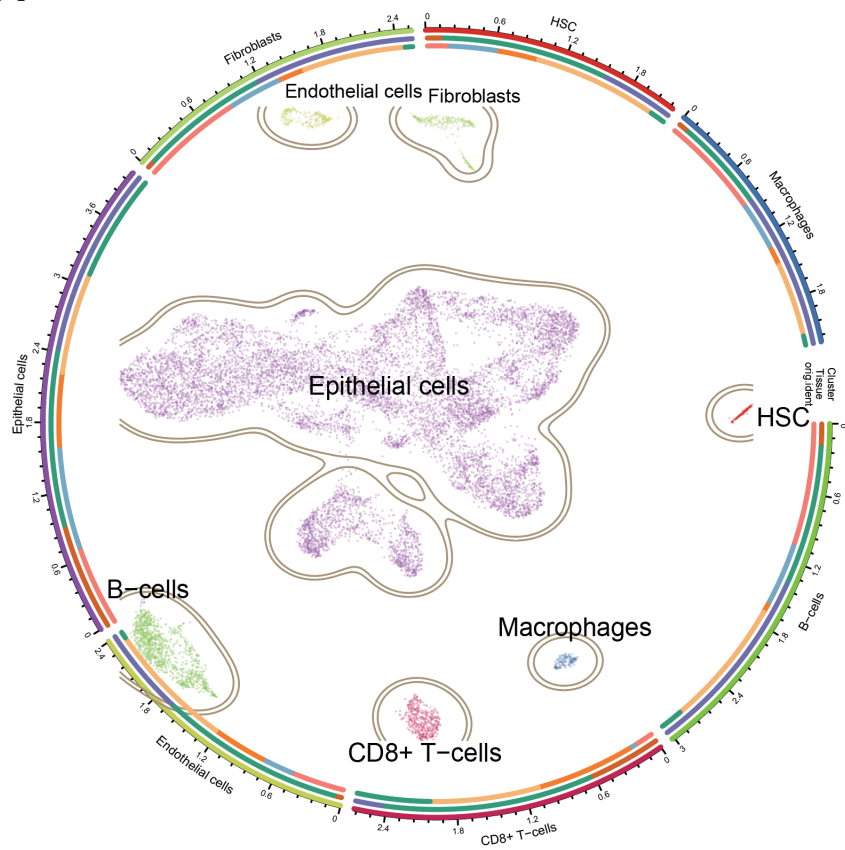**B**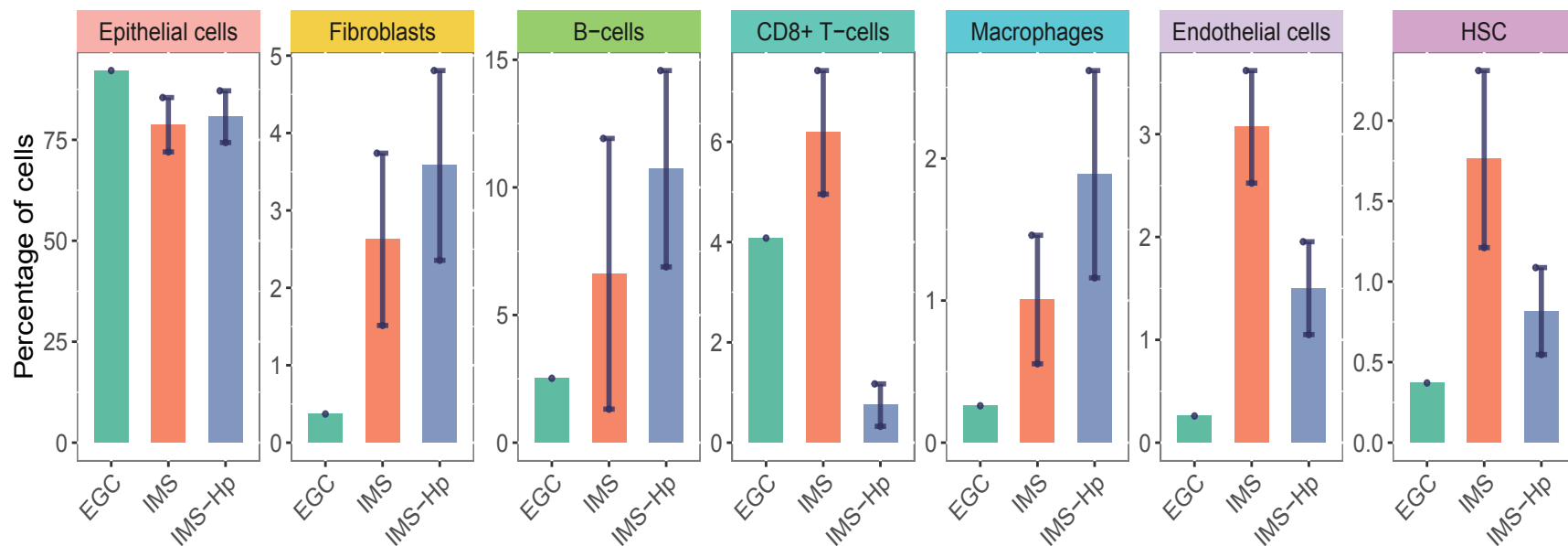**C**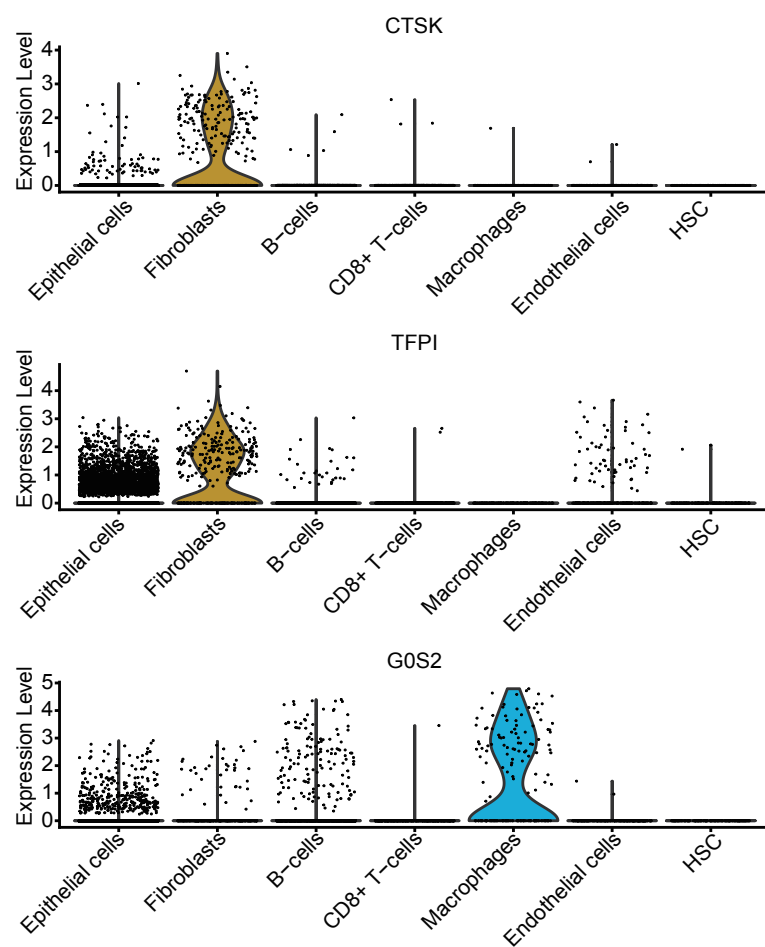

Supplement: Supplementary file 5 — Figure S5: Heterogeneity of gastric cancer in single‐cell data. [file CNR2-9-e70511-s009.pdf]

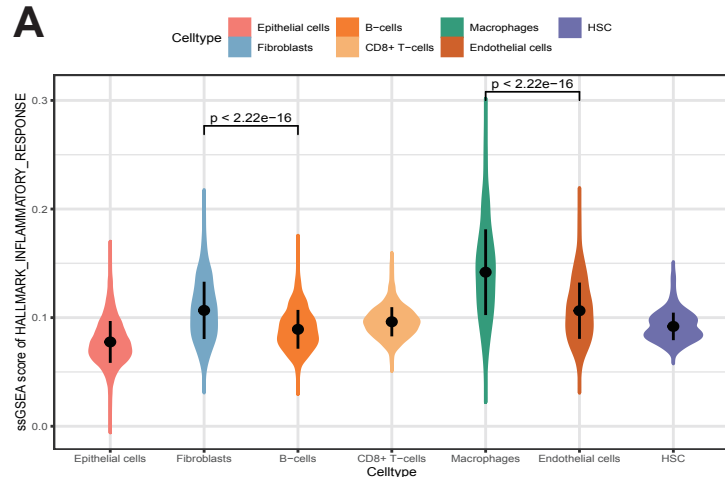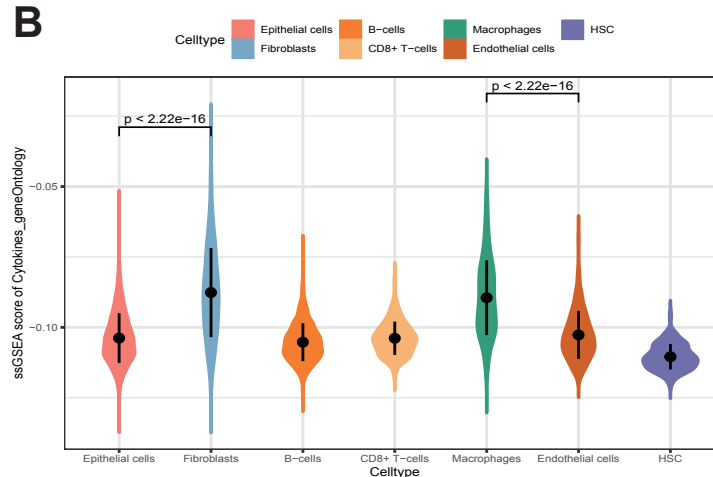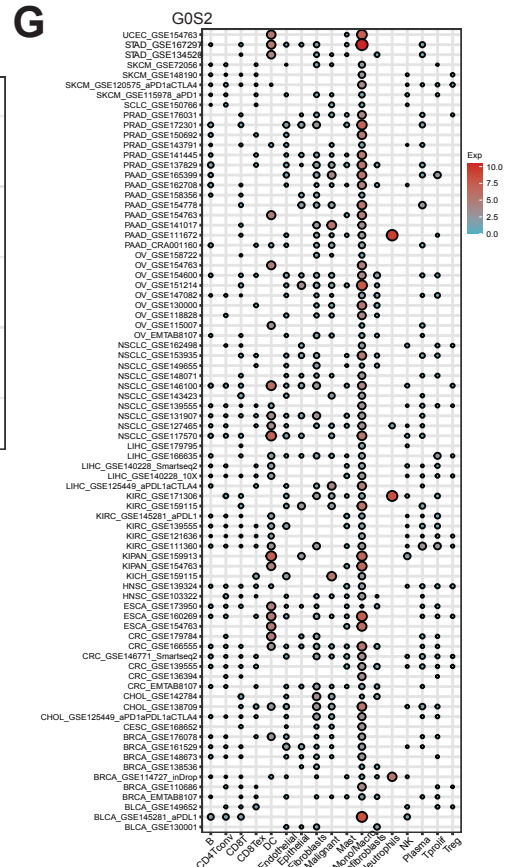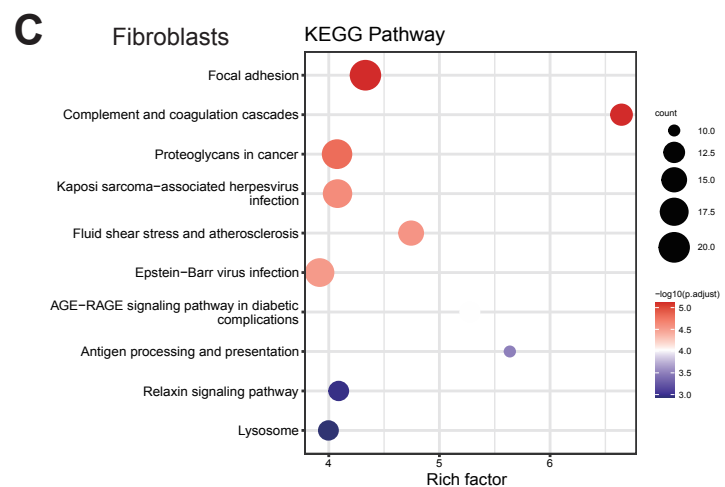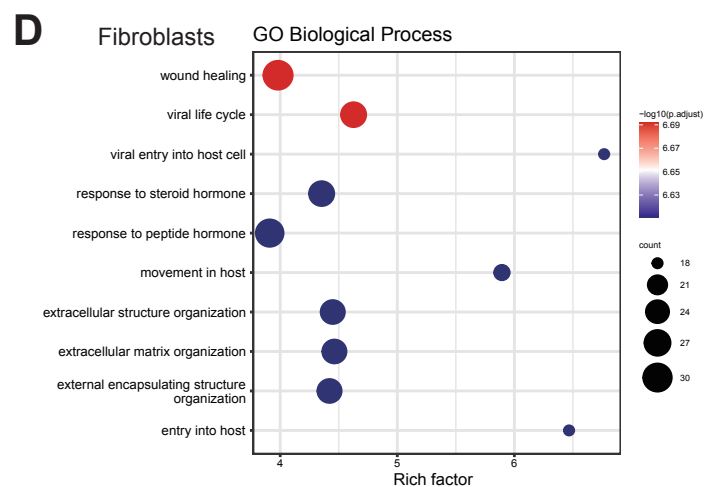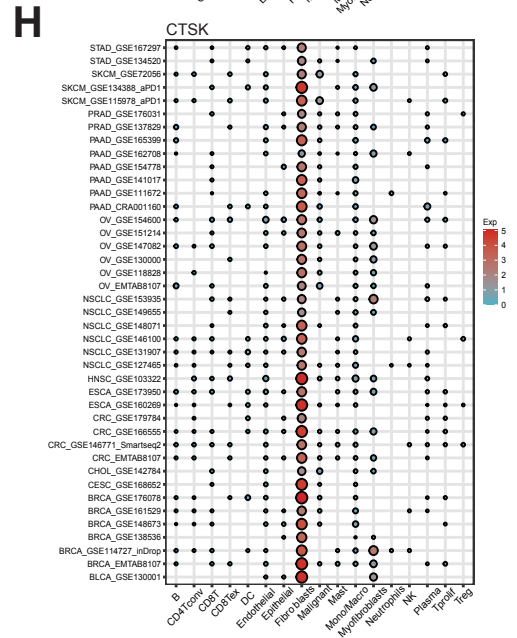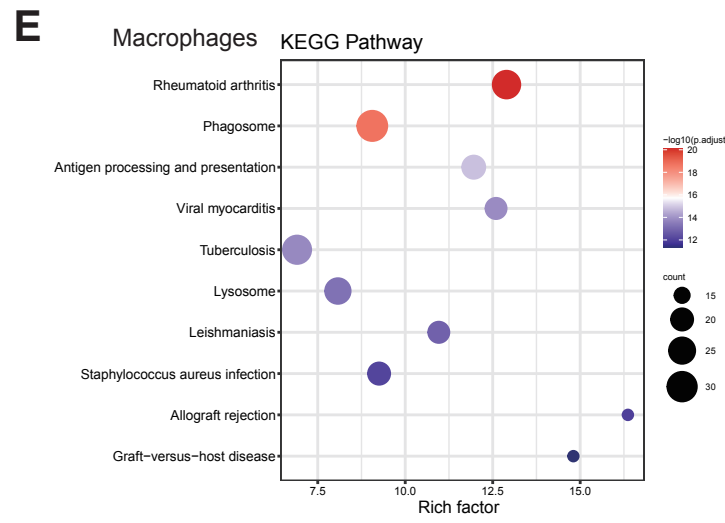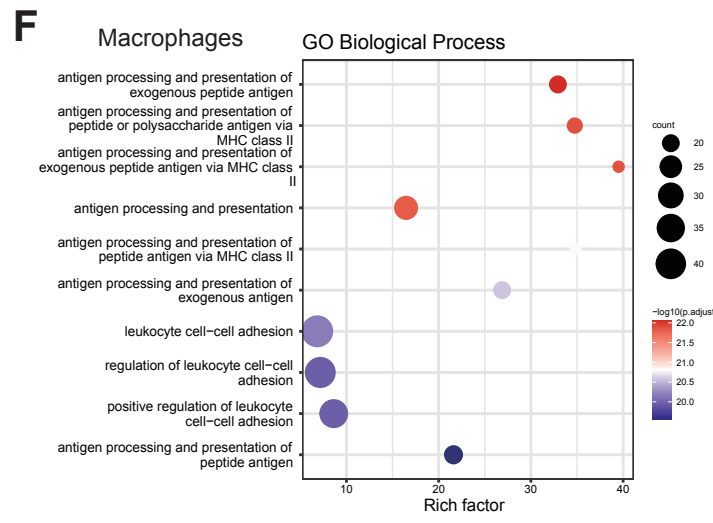

Supplement: Supplementary file 6 — Figure S6: Expression profiles of key prognostic factors across cell types and pan‐cancer. [file CNR2-9-e70511-s004.pdf]
